# Loneliness as an active ingredient in preventing or alleviating youth anxiety and depression: a critical interpretative synthesis incorporating principles from rapid realist reviews

**DOI:** 10.1038/s41398-021-01740-w

**Published:** 2021-12-10

**Authors:** Eiluned Pearce, Pamela Myles-Hooton, Sonia Johnson, Emily Hards, Samantha Olsen, Denisa Clisu, Sarah M. A. Pais, Heather A. Chesters, Shyamal Shah, Georgia Jerwood, Marina Politis, Joshua Melwani, Gerhard Andersson, Roz Shafran

**Affiliations:** 1 Division of Psychiatry, University College London, London, UK; 2 Great Ormond Street Institute of Child Health, University College London, London, UK; 3 Camden and Islington NHS Foundation Trust, London, UK; 4 Department of Psychology, University of Bath, Bath, UK; 5 Division of Psychology and Language Sciences, University College London, London, UK; 6 Department of Behavioural Sciences and Learning, Department of Biomedical and Clinical Sciences, Linköping University, Linköping, Sweden

**Keywords:** Depression, Scientific community

## Abstract

Loneliness is a relatively common problem in young people (14–24 years) and predicts the onset of depression and anxiety. Interventions to reduce loneliness thus have significant potential as active ingredients in strategies to prevent or alleviate anxiety and depression among young people. Previous reviews have focused on quantitative evidence and have not examined potential mechanisms that could be targets for intervention strategies. To build on this work, in this review we aimed to combine qualitative and quantitative evidence with stakeholder views to identify interventions that appear worth testing for their potential effectiveness in reducing loneliness, anxiety and depression in young people aged 14–24 years, and provide insights into the potential mechanisms of action. We conducted a Critical Interpretative Synthesis, a systematic review method that iteratively synthesises qualitative and quantitative evidence and is explicitly focused on building theory through a critical approach to the evidence that questions underlying assumptions. Literature searches were performed using nine databases, and eight additional databases were searched for theses and grey literature. Charity and policy websites were searched for content relevant to interventions for youth loneliness. We incorporated elements of Rapid Realistic Review approaches by consulting with young people and academic experts to feed into search strategies and the resulting conceptual framework, in which we aimed to set out which interventions appear potentially promising in terms of theoretical and empirical underpinnings and which fit with stakeholder views. We reviewed effectiveness data and quality ratings for the included randomised controlled trials only. Through synthesising 27 studies (total participants *n* = 105,649; range 1–102,072 in different studies) and grey literature, and iteratively consulting with stakeholders, a conceptual framework was developed. A range of ‘Intrapersonal’ (e.g. therapy that changes thinking and behaviour), ‘Interpersonal’ (e.g. improving social skills), and ‘Social’ Strategies (e.g. enhancing social support, and providing opportunities for social contact) seem worth testing further for their potential to help young people address loneliness, thereby preventing or alleviating depression and/or anxiety. Such strategies should be co-designed with young people and personalised to fit individual needs. Plausible mechanisms of action are facilitating sustained social support, providing opportunities for young people to socialise with peers who share similar experiences, and changing thinking and behaviour, for instance through building positive attitudes to themselves and others. The most convincing evidence of effectiveness was found in support of Intrapersonal Strategies: two randomised controlled studies quality-rated as ‘good’ found decreases in loneliness associated with different forms of therapy (Cognitive Behavioural Therapy or peer network counselling), although power calculations were not reported, and effect sizes were small or missing. Strategies to address loneliness and prevent or alleviate anxiety and depression need to be co-designed and personalised. Promising elements to incorporate into these strategies are social support, including from peers with similar experiences, and psychological therapy.

## Introduction

Loneliness can be defined as a perceived mismatch between actual and desired quantity or quality of relationships, arising through the interplay of predisposing (individual, situational and cultural) and precipitating factors (e.g. life transitions such as bereavement or moving to university) [1]. If the situation remains unchanged, chronic loneliness may develop: an intrinsically aversive and stable state associated with the inability to develop satisfying social relationships over a sustained period, linked with physical and psychiatric consequences [2]. Given that many intervention studies to date do not distinguish between transitory and chronic loneliness, we explore loneliness in general here.

Loneliness is relatively prevalent amongst 16–25-year olds [3], and longitudinal studies demonstrate that loneliness during childhood increases risk of depression and emotional symptoms up to 24 years later [4, 5]. A meta-analysis demonstrated a negative feedback loop between adolescent social anxiety and loneliness over time [6: across studies *r* = 0.1–0.3]. In a clinical sample of adolescents, loneliness measured at 9 months post-baseline was found to mediate an indirect relationship between baseline social anxiety and suicidal ideation measured at 18 months post-baseline [7]. Moreover, loneliness was found to be a significant mediator in the relationship between anxiety and depression in both a school-based sample and youth receiving residential treatment [8]. Despite loneliness, social anxiety and depressive symptoms being interrelated, they are statistically and experientially distinguishable [9, 10].

Three recent systematic reviews of quantitative studies have included in their scope investigation of loneliness interventions in the context of mental health and/or young people. First, Ma et al. [11] examined randomised controlled trial (RCT) interventions for reducing loneliness in individuals of all ages experiencing mental ill-health, and found one intervention for female undergraduates with depression [12] and one for high school students with social anxiety [13]. Second, Loades et al. [14] reviewed two RCTs of (i) a mentorship programme for 12–15-year olds experiencing victimisation [15], and (ii) a school-based intervention for 15–19-year olds involving either a one-tier intervention comprising class activities and student mentors, or a two-tier programme that additionally involved a staff mental health support team [16]. Third, a meta-analysis found that a range of interventions reduced loneliness in youth aged 25 years or younger across diverse samples including those with anxiety or depression [17].

However, these recent reviews only include quantitative evidence and do not focus on anxiety and depression despite the clear links between these internalising problems and loneliness. Moreover, these previous reviews do not include investigations of potential mechanisms of action. Thus, while interventions to reduce loneliness have potential as active ingredients in strategies to reduce depression and anxiety among young people, currently we do not have robust evidence as to which strategies have potential to be effective and in which contexts, and why. Loneliness interventions in the area of mental health is an emerging field, and insufficient numbers of adequately powered and appropriately designed studies means the quantitative evidence is limited. Consequently, identifying promising approaches also requires qualitative evidence to provide a more nuanced and experiential perspective to complement the quantitative work [10]. Additionally, third sector organisations are active in addressing loneliness, and new insights can be gained from incorporating their practical service-led perspectives. Synthesis of quantitative, qualitative and grey literature evidence, together with consideration of mechanisms and pathways underpinning potential interventions, and stakeholder views regarding intervention acceptability and potential usefulness, is needed to provide convergent support for which strategies are worth testing for their potential to reduce loneliness in young people, and therefore prevent or alleviate anxiety and depression.

To fill this gap, we conducted a Critical Interpretive Synthesis (CIS) [18, 19], in order to iteratively critique and integrate multidisciplinary and multi-method evidence, generate overarching conceptual constructs and form a new, critically-informed theoretical framework. CIS is a robust method that draws on both systematic review and qualitative methods to identify links between constructs already reported in the literature, and higher-level overarching ‘synthetic’ constructs that draw together different sources of evidence. The aim is to generate theory with strong explanatory power [18]: that is, which makes clear and testable predictions based on observations rather than assumptions, including about causal mechanisms. For instance, in this review we aimed to generate a theoretically driven framework that allows hypotheses to be proposed about what interventions to reduce loneliness might work for whom, and why. The overarching synthetic constructs in CIS are generated through critically exploring how the authors of included quantitative and qualitative studies have conceptualised and constructed the phenomenon under consideration, and questioning the assumptions made in different empirical and theoretical approaches. This review method is particularly useful in optimising the usefulness of the limited data available in separate research fields, by meaningfully integrating cross-disciplinary, cross-method and cross-sector evidence to yield new holistic insights. This approach takes an iterative but systematic approach to question formulation, searches and selection of evidence, with the latter being based on relevance to the research question rather than quality. There is an active questioning of underlying assumptions in the literature and a conceptual framework is developed through a dialectic process between the evidence and theory. To complement the CIS approach, we also incorporated principles from Rapid Realist Review (RRR) [20] by engaging stakeholders with academic and/or lived experience expertise, ensuring relevance to policy and practice.

A number of different classifications of loneliness interventions have been proposed previously. For instance, in a meta-analytic review of 50 studies that together spanned all age groups, Masi et al. [21] adopted a classification comprising four primary intervention strategies, which they identified from previous qualitative reviews: (i) improving social skills, (ii) enhancing social support, (iii) increasing opportunities for social contact, and (iv) addressing maladaptive social cognition. More recently, Mann et al.’s [22] scoping review focused on individuals with mental health problems, and categorised ‘direct’ interventions that targeted loneliness and concepts related to social relationships (as opposed to broader wellbeing interventions, which might also impact on loneliness ‘indirectly’) into four broad groups: (i) changing cognitions (e.g. cognitive behavioural therapy or reframing), (ii) social skills training and psychoeducation (e.g. family psychoeducation therapy), (iii) supported socialisation or having a ‘socially-focused supporter’ (e.g. peer support groups, social recreation groups), and (iv) wider community approaches (e.g. social prescribing and asset-based community development approaches) [11, 22]. Mann et al. classified specific interventions based on the main approach used, but point out that these categories are not mutually exclusive. This latter typology was adopted by Ma et al. [11] in their review of RCTs described above. Eccles and Qualter [17] divided interventions for individuals under 25 years into (i) social skills, (ii) social interaction, (iii) social and emotional skills, (iv) enhanced social support, (v) psychological intervention, (vi) learning new skills, (vii) other, as well as noting whether delivery was individual or group, and using technology or not.

In our conceptual framework we aimed to provide insights into promising approaches that should be targeted for further development and testing by answering the research questions: (i) in which ways and in which contexts does addressing loneliness appear to have potential to prevent and/or improve anxiety and depression in young people and why, and, (ii) in which ways and which contexts and for whom, does addressing loneliness appear not to work, and why? Consequently, in contrast to the previous classifications of loneliness interventions described above, we not only aimed to provide a typology of interventions, but also a conceptual model that additionally incorporates a classification of context (who the intervention works or does not work for) and mechanism (why the intervention works or does not work). As is inherent in the CIS approach, we aimed to question the relevance of previous typologies of loneliness interventions to this particular age group and from the perspective of preventing and alleviating anxiety and depression. We focus on the 14–24 age group in line with the Wellcome Trust’s mental health programme strategy [23], since half of all lifetime cases of mental health problems start by age 14 and 75% by age 24 years, meaning that this is a critical period for potential intervention [24].

## Methods

### Search strategy

The aim of the searches was to identify interventions to address loneliness in 14–24-year olds that also related to anxiety or depression: for example because the intervention targeted participants already experiencing depressive symptoms or diagnosed with depression, or because the measured outcomes included anxiety or depression as well as loneliness. We began with an ‘a priori’ search strategy focusing on interventions to address loneliness in young people that either also measured anxiety and/or depression (to identify prevention strategies and their mechanisms) or for which the sample comprised young people experiencing anxiety and/or depression (to identify treatment strategies and their mechanisms) [25]. Studies conducted outside of the UK were included as long as they were reported in English.

The initial searches were followed by further iterations of targeted searches [18] for terms raised by the Lived Experience Advisory Group (LEAG) and academic experts, such as ‘stigma’. Initial searches used modified search terms from [14] to update quantitative literature published subsequently to [14], and to search for qualitative studies (see Supplementary Materials for details of searches, including for grey literature; search terms are given in Supplementary Table S1). We chose to update the search for quantitative papers rather than conducting searches for all published studies from all dates because the previous review had been published within 6 months of our searches, and related specifically to loneliness and mental health in young people. Moreover, unlike in ‘standard’ systematic reviews, the aim of the CIS approach is not to identify and include all relevant literature but to reach ‘theoretical saturation’, that is, to include enough literature from a range of sources (including from prior reviews) to ensure that all key themes and concepts are covered [18]. The searches for published qualitative and grey literature were novel searches that included all dates and were not updates of previous reviews.

**Fig. 1:**
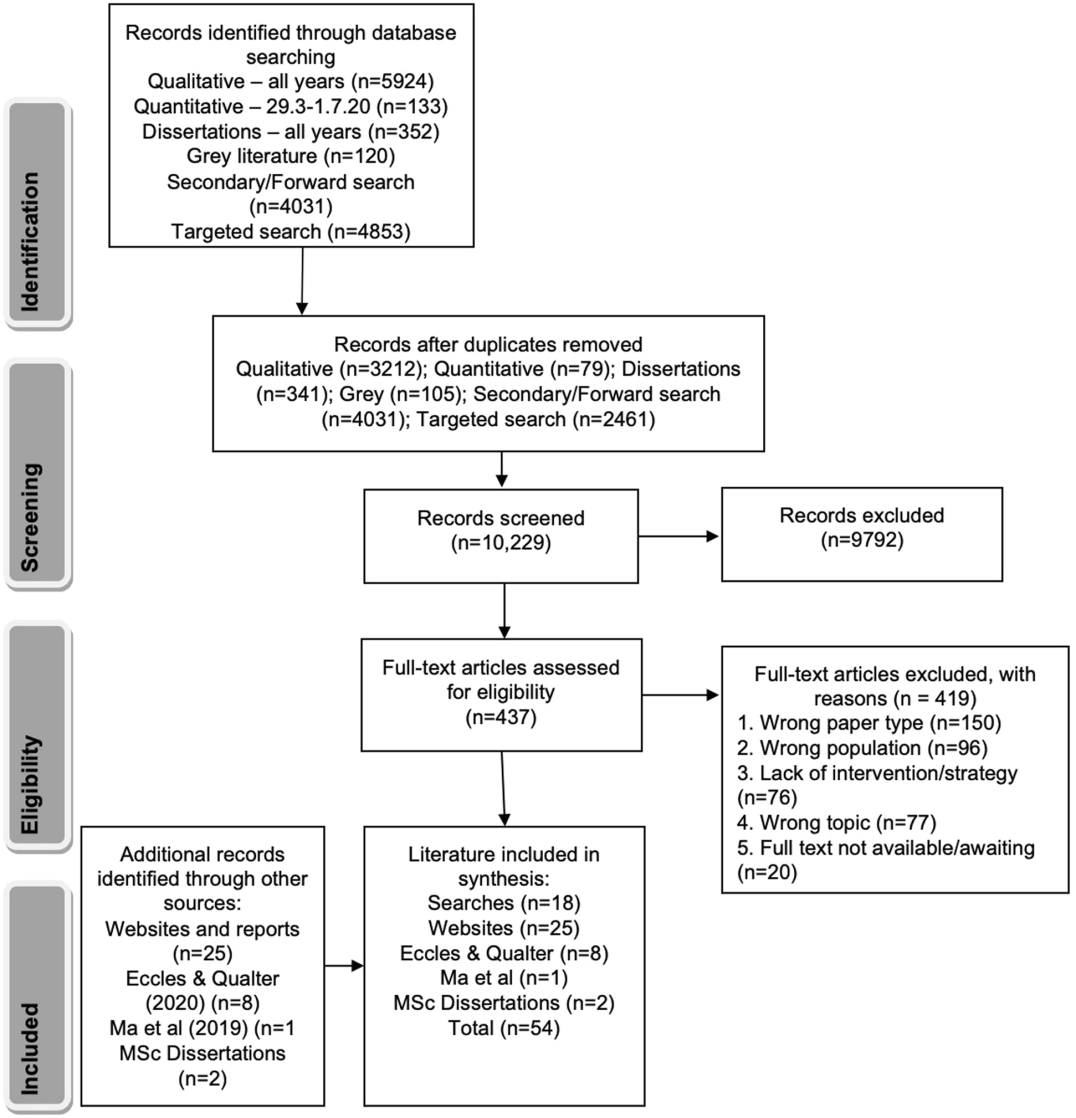
PRISMA flow diagram of included and excluded evidence. The number (*n*) of records identified, screened by (i) title and abstract and (ii) full text, excluded (with reasons for exclusion) and included in the synthesis from different sources are provided.

Due to the small number of studies found in test searches, we widened the search and inclusion criteria to incorporate ‘mental health’ (including wellbeing) more generally. For inclusion, quantitative and qualitative studies required: loneliness as a primary or secondary outcome in the context of anxiety, depression, or ‘mental health’ (broadly defined to include wellbeing), publication in English in a peer-reviewed journal, a mean sample age within the 14–24 years range, and that the study included an intervention or coping strategy addressing loneliness. Grey literature was included along similar lines, without the publication criterion. Articles were excluded that did not: investigate loneliness, depression, anxiety, mental health or wellbeing, fit the age range, or include an intervention or strategy addressing loneliness. The Preferred Reporting Items for Systematic Reviews and Meta-Analysis (PRISMA) statement [26] was followed (Fig. 1) and the review protocol was registered on the PROSPERO database [CRD42020197953].

### Integrating the views of experts

A LEAG of 18–24-year olds with lived personal experience of loneliness and mental ill-health (in the recruitment material mental ill-health was described as anxiety, depressive symptoms, mental distress, low mood, or excessive worry) (*n* = 18) were recruited by circulating an advert through the UKRI Loneliness & Social Isolation, Emerging Minds and SMaRteN mental health research networks (http://mentalhealthresearchmatters.org.uk/networks/), the McPin Foundation (a mental health research charity) and the Birmingham University Institute of Mental Health Youth Advisory Group. These networks and organisations facilitate patient and public involvement in research and young people were invited to be stakeholder consultants in this research project; the young people involved were not research participants. Individuals interested in being involved were asked to complete an online expression of interest form. All young people who expressed an interested in being involved were invited to join the group: 19 young people expressed an interest but one dropped out before the first meeting for personal reasons. Due to time constraints, we did not recruit through non-UK networks, but three LEAG members were international students studying in the UK. We did not collect standard demographic information from LEAG members as they were providing consultation and were not research participants, but the expressions of interest form invited applicants to provide any information they thought would be relevant given our aim to recruit a diverse group. The information volunteered indicated that several members identified with a non-binary gender, and a range of sexual orientations and ethnic groups were represented. Several members identified as having Autism Spectrum Disorder (ASD) and several were care leavers. The majority were students or recent graduates from UK universities. At the same time as expressing an interest in joining the LEAG, individuals could also volunteer to become ‘Lived Experience Leads’, who would take a more active role in creating the dissemination outputs required by our funder. About half of the group expressed an interest in the ‘Lead’ role and we chose four individuals who would provide a variety of perspectives given their demographic characteristics and backgrounds, and who had experience relevant to research, and creating videos, infographics and lay summaries. All LEAG members were invited to attend three online meetings (2 h each) across the 4 months of the project. In preparation for the second and third meetings they were asked to review, respectively, (i) the initial conceptual framework and (ii) the dissemination materials including drafts of the lived experience commentary included in this paper, and to provide feedback during the meetings, which were facilitated by the four Leads. The Leads provided additional comments on the search protocol, the developing conceptual framework and this research paper outside of the meetings, as well as creating the lived experience commentary on this paper, a lay summary, an infographic for policy makers and a short video aimed at 14-year olds explaining the research findings. All LEAG members received Patient and Public Involvement payments of £20 per hour, reflecting standard UK rates at the time.

Written and verbal feedback was sought on the search protocol from interdisciplinary experts (co-investigators of the Loneliness and Social Isolation in Mental Health Research Network: see Acknowledgments for the diverse disciplines represented) and four Lived Experience Leads selected from the LEAG. Three further academic experts were consulted for additional published or unpublished work. The authors of this paper include clinicians (clinical psychology and psychiatry) who also conduct academic research, an evolutionary anthropologist, and people with lived experience of mental ill-health and/or loneliness. This paper reflects a process of discussion between these diverse perspectives that was ongoing throughout the research project.

### Study selection

#### New searches

Twenty-two potential studies were found and four were excluded (see Supplementary Materials for screening details), leaving 18 (Fig. 1 and Table 1; ref. [27] is a Ph.D. thesis). Thirty-three papers related to built environment interventions for youth mental health provided by an expert did not meet inclusion criteria: these papers were a subset from a wider systematic review search looking at built environment interventions for improving mental and physical health in children and young people. Although six of these papers included concepts related to loneliness (1 paper related to ‘community belonging’, 1 to ‘sense of community’, and 4 to ‘social cohesion’), none of the papers related to loneliness per se and were therefore excluded.

**Table 1:** Study details for the 18 included papers from the new searches.

Author (year), country	Study design	Sample	*N* in sample completing measurement at all time-points (*n* male, % male)	Age range (Mean, SD)	Measures	Intervention/ strategy and duration	Delivery method/ format	Comparison condition	Main findings
Agadullina et al. (2020) Russia [52]	Experimental, randomised controlled study	Psychology first-year students No power calculation reported.	77 (23, 30%) 40: intervention 37: control	n.s. (19.9, 0.13)	Social networks use questionnaire; Motives for Social Network Sites use (social inclusion—3 items; meeting people—3 items, maintaining relationships—3 items); Multidimensional inventory of loneliness experience (MILE) [77] (social loneliness—4 items; emotional loneliness—4 items; negative attitudes—7 items; positive attitudes—7 item). Pre, mid, post.	Quit social network sites (SNS) (4 weeks)	Individual, tech	Randomly divided into two groups: intervention or control condition (continued to use SNS as usual).	Quitting SNS did not change either feeling of social or emotional loneliness. Feelings of social and emotional loneliness did not depend on students’ positive/negative attitude towards being alone when looked at over time. Effect sizes not reported.
	Semi-structured interviews	Psychology first-year students	18 (n.s.)	n.s.	Randomly selected participants from the treatment group took part in semi-structured interviews. (average 25 min). Post.	Quit social network sites (SNS) (4 weeks)	Individual, tech	Randomly selected from the intervention group.	Two main themes: 1— negative feelings; 2—positive feelings of quitting SNS. Negative subthemes: adaptation—in first week hard to quit SNS; loss of information sources and contact with people. After end of adaptation period negative emotions often replaced by positive ones. Positive subthemes: previous positive experience of quitting SNS; improved offline communication; more time for reading, study and self-development. Sample said they did not feel lonely because of often communicating with peers and classmates personally, allowing them to realise the need for maintaining relations and communication. Quitting SNS led some to an understanding that spending a lot of time on SNS can be a problem and an obstacle to development.
Bernhold (2020), USA [66]	Correlational study	Undergraduate students	401 (n.s., 28.9%)	n.s. (19.77, 1.55)	Grandchildren Received Affection Scale; Shared Family Identity; Future Time Perspective Scale; Revised UCLA Loneliness Scale (3-item^a^) [78]; Centre for Epidemiological Studies—Depression Scale; Short Form Perceived Stress Scale (6-item).	None	N/A	N/A	Grandparents’ affectionate communication toward grandchildren was indirectly related to reduced loneliness via increased shared family identity, but only for grandchildren who viewed their grandparents’ future as expansive. Effect size not reported.
Dagan and Yager (2019) Israel [40]	Case study	2 × case studies (1 male—32 and 1 female—24 with complex PTSD). Only female case study used.	1 (0, 0%)	24 (n/a, n/a)	None	Psychodynamic psychotherapy and developing new relationships following inpatient treatment and medication.	Individual, non-tech	N/A	Alleviating loneliness through the development of relationships made progress in psychotherapy easier for her.
dos Santos et al. (2020), Brazil [58]	Secondary cross-sectional analysis from the National School-based Health Survey (PeNSE in Portuguese) in Brazil	Students from public and private schools. Representative sample but no power calculations reported for this analysis.	102,072 (49,290, 48.3%)	11–19 (14.33, 1.06)	Self-report electronic questionnaires assessed feelings of loneliness^b^ (1-item), number of friends (1-item), Physical Activity Indicators Questionnaire, physical exercise (1-item), leisure time physical activity (1-item), active commuting (1-item).	None	N/A	N/A	The greater the weekly time accumulated in physical education classes, the lower the likelihood of feeling loneliness and having few friends for both sexes. A similar association pattern was observed between leisure-time physical activities and social isolation indicators. Those with longer active commutes had a greater likelihood of feeling loneliness for both sexes, and greater likelihood of having few friends for girls (this was not significant for boys). Effect size not reported.
Gold et al. (2019), USA [47]	Quantitative surveys	Medical students	18 (3, 17); 30 undertook the intervention. Power calculation indicated *n* = 10 for 80% power but loneliness analysis based on *n* = 6	n.s.	Modified Emotional Self-Awareness Scale (18-item); Modified Interpersonal Fulfilment Index (21-item); Revised UCLA Loneliness Scale (20-item: [79]). Pre and post. 9 × 5-point Likert scale questions evaluating programme Post.	Facilitated reflective social support groups (1.5 h every 2 weeks for 6 months)	Group, non-tech	N/A	Students reported improvement in wellbeing, enhanced self-awareness, and ability to empathise, and promoted connection. In a pilot subsample (*n* = 6), there was a decrease in loneliness (20%) with negligible change in empathy and wellbeing scores. Effect sizes not reported.
	Qualitative questions in surveys	Medical students	18 (3, 17)		1 open-ended qualitative question assessing expectations and goals for group participation. Pre. 4 qualitative questions assessing what students derived from groups. Post.	Facilitated reflective social support groups (1.5 h every 2 weeks for 6 months)	Group, non-tech	N/A	Main themes: overcoming impostor syndrome (realising others have similar experiences and one is not alone) through shared experience; improved connection with others (decreased loneliness); exposure and tolerance to diverse perspectives; insight into importance of self-care; increase in self-awareness and acceptance.
Hillier et al. (2018), USA [54]	Pre-post test	University students with ASD	25 (n.s., n.s.). Baseline sample: 52 (51, 98%). No power calculations reported.	Baseline: 18–28 (20.9, n.s.)	Rosenberg Self-Esteem Scale, UCLA Loneliness Scale^4^ (20-item^c^), Counselling Center Assessment of Psychological Symptoms-34 Scale. Pre and post. Acceptability, social relevance and usefulness were assessed using a brief questionnaire. Post.	Support group: addressing common challenges among university students with ASD (1 h weekly for 7 weeks). Includes sessions on ‘interpersonal communication and relationships’ and ‘Working in groups’.	Group, non-tech	N/A	Significant reductions in feelings of loneliness and general anxiety, and significant increase in self-esteem. Effect sizes not reported.
	Focus groups	University students with ASD	26 (n.s., n.s). Baseline sample: 52 (51, 98%)	Baseline: 18–28 (20.9, n.s.)	Focus groups discussed experiences as a university student and the impact of the programme on academic behaviours and social skills aimed to examine functional changes in academic and social skills. (3–5 participants per group lasting 45–75 min). Post.	Support group: addressing common challenges among university students with ASD (1 h weekly for 7 weeks). Includes sessions on ‘interpersonal communication and relationships’ and ‘Working in groups’.	Group, non-tech	N/A	Five themes from focus-group analysis reflected positive impact on skills and coping: executive functioning; goal setting; academics and resources; stress and anxiety; and social (programme helped interactions with other students within and outside the group). Feelings of loneliness decreased, and some participants mentioned how meeting other ASD students had been beneficial.
Horgan et al. (2013), Ireland [36]	Pre-post test	University students with self-reported depressive symptoms	16 (13, 81%). Baseline sample: 118 (75, 64%). No power calculations reported but differences were not expected by the authors to be significant due to small sample size.	Baseline:18–24 (21, n.s.)	Centre for Epidemiological Studies—Depression includes 1 loneliness item^d^ Pre and post.	Website ‘losetheblues’ for 18–24 year-olds experiencing depressive symptoms, including a forum offering an opportunity for peer support (6 weeks).	Individual/ Group tech	N/A	No statistically significant effect of intervention on loneliness or depressive symptoms, but 86% attrition rate.
	Analysis of forum posts	University students with self-reported depressive symptoms	17 (n.s., n.s.) (10 of sample had completed CES-D at post-test)		Forum posts (*n* = 53) were analysed from 17 users (3 months).	Website ‘losetheblues’ for 18–24 year-olds experiencing depressive symptoms, including a forum offering an opportunity for peer support (6 weeks).	Individual/ Group tech	N/A	Forum posts showed main difficulties were loneliness and perceived lack of socialisation skills. Some students found the website a useful place for sharing, offering, and receiving emotional and informational support.
Kivijärvi et al. (2019), Finland [55]	Two-arm, quasi-experimental randomised controlled study	Young adults not in employment or education	Phase 2 (feedback survey): 26 (12, 46%^e^). Phase 3 (quantitative, qualitative survey): 107 (40, 37% ^e^) (41 intervention group; 106 control group). Baseline sample: 147 (66, 45%). No power calculations reported.	Baseline: 16–30 (23.07, 3.30^e^)	Survey (incl. mainly validated measures that are not stated) on living conditions, quality of life (QoL), social relations, trust, capabilities, service use, living habits, and a 12-item version_e_ of the Revised UCLA Loneliness Scale. and Post. The WHO Quality of Life Brief Instrument; frequency of loneliness (1 item). Pre and post. Feedback survey Post.	Moderated anonymous online groups ‘MAOG’ to promote the wellbeing of participants by providing a low threshold and secure space to discuss matters of importance to them, to be positively recognised, and to alleviate feelings of loneliness (9 weeks).	Group, tech Group	Control condition: did not receive MAOG	No significant changes in QoL or loneliness observed among the participants or between the two arms of the study. When looking at both QoL and loneliness, levels remained the same for the control group and for active discussants. For passive discussants there was a slight drop in QoL and a rise in the level of loneliness. Acceptability was highest among those with poor QoL and loneliness and who had difficulties in engaging with face-to-face groups. Results on viability were ambivalent. Professionals responsible for moderating the online groups were relatively satisfied. No effect sizes reported.
	Group interviews and workshops, scrutiny of online discussion threads, evaluation of online group activities, and review of open-ended questions in feedback questionnaire	Young adults not in employment or education	Phase 1 (workshops): 16 (n.s.). Phase 2: 41 online discussions (20, 49% ^e^). Phase 3: 26 open-ended questions in feedback questionnaire (12, 46% ^e^)	Baseline: 16–30 (23.07, 3.30 ^e^)	Three phases of data collection: analysis of comments from group interviews and workshops – pre (*n* = 16) + youth workers (*n* = 30); scrutiny of ways in which discussants participated and content/tone of posts in online discussions – during (*n* = 41) + youth workers (n = 6); review of responses to open-ended questions in feedback questionnaires – post (*n* = 26).	Moderated anonymous online groups ‘MAOG’ to promote the wellbeing of participants by providing a low threshold and secure space to discuss matters of importance to them, to be positively recognised, and to alleviate feelings of loneliness (9 weeks)	Group, tech Group	N/A	During workshops the most frequently mentioned need for help was related to peer sociability, with the need for moderation, safety, a positive atmosphere and anonymity also emphasised. Most acceptable themes of the online discussions were related to social relationships and loneliness. The feedback survey found that for some participants, the online groups functioned as a forum for disclosure and testing one’s ideas among a peer group – often in relation to the themes of loneliness or mental health.
Lim et al. (2019), Australia [41]	Single intervention study, pilot proof-of-concept study	Lonely young people with or without a diagnosis of social anxiety disorder (SAD)	20 (n.s., n.s.). 9 (5, 55.56%) with SAD. 11 (6, 54.55%) students with no mental disorder. No power calculation reported but this was explicitly framed as a proof-of-concept pilot study.	18–23 SAD (21, 1.41); no mental disorder (20.36, 0.52)	Revised UCLA Loneliness Scale (20-item: [79]); straightforwardly worded items of the Social Interaction Anxiety Scale (S-SIAS); Centre for Epidemiological Studies—Depression (CES-D). Pre, post (minimum 33 days of +connect), follow-up (3 mo.).	Digital smartphone app (+Connect) delivers positive psychology designed to improve relationship quality. (6 weeks)	Individual, tech	Non-clinical student sample undertook the same procedure	UCLA-LS and S-SIAS decreased from baseline to follow-up for SAD group. CES-D decreased from baseline to post but scores appeared to regress toward their baseline at follow-up. UCLA-LS, S-SIAS, and CES-D scores decreased from baseline to post-intervention and follow-up for control student group. Large effect sizes reported for decrease in loneliness for both groups together and separately: Cohen’s *d* = ~1. Authors state that because their aim was to be descriptive, they do not provide tests of between-group differences.
	Semi-structured interviews	Lonely young people with or without a diagnosis of social anxiety disorder (SAD)	20 (n.s., n.s.). 9 (5, 55.56%) with SAD. 11 (6, 54.55%) students with no mental disorder	18–23 SAD (21, 1.41); no mental disorder controls (20.36, 0.52)	Semi-structured interviews. Post (minimum 33 days of +connect).	Digital smartphone app (+Connect) delivers positive psychology designed to improve relationship quality. (6 weeks)	Individual, tech	Non-clinical sample undertook the same procedure	75% of participants reported at least one positive outcome from using the app. These primarily included increased positive affect and improved social interactions. Four participants (2 SAD, 2 controls) reported no positive outcomes from using the app. No participants reported negative outcomes from using the app. Positive outcomes driven by three processes: reflection, learning, real-life application.
Mahdavi et al. (2020), Iran [46]	Single-case design	Runaway adolescent girls	4 (0, 0%). No power calculations reported.	15–16 (15.75, n.s.)	UCLA Loneliness Scale (20-item: [80]); Emotion Regulation Difficulty Questionnaire. Pre, post, follow up (1 mo.).	Bi-weekly sessions of compassion-based or schema-based training (8 weeks)	Individual, non-tech	Assigned to compassion-based or schema-based training using purposive sampling.	Both trainings reduced loneliness and emotional dysregulation, but compassion-based techniques were reported to be more effective based on changes in individual scores. No statistical tests are reported.
Park (2012), USA [27]	Content analysis	Hospitalised children and adolescents with physical illnesses	10 (4, 40%)	13–18 (15.8, n.s.)	Semi-structured interviews post music therapy sessions—30–45 min. Post.	Music therapy (1–35 sessions)	Group, non-tech	N/A	Participants described music therapy as helping them to relieve and express their pain, anxiety, depression, loneliness, and helping them to bond with their family.
Rew (2000), USA [65]	Focus group and in-depth individual interview	Homeless adolescents	32 (18, 56.25%): Subsample: 10 (6, 60%)	16–23 (19.1, n.s.) subsample: 15–23 (19.2, n.s.)	Focus groups (6–10 participants per group × 4 groups = 32 participants); Subsample - individual semi-structured interviews = 10.	None	N/A	N/A	The majority (*n* = 26, 81%) identified two primary coping strategies for loneliness: being with friends, and having a dog for a companion.
Rice, et al. (2020a), Australia [38]	Participatory design	Young people with lived experience of Social Anxiety Disorder	16 (n.s.)	n.s.	4 × forums (1–2 h) of young people with SAD provided feedback on the development of the social media based intervention.	Development of 12-week ‘Entourage’ intervention targeting cognitive and behavioural components of SAD.	Development: forums - group, non-tech Intervention – individual with peer to peer online social networking, tech.	N/A	A digital mental health intervention was developed incorporating the experiences of young people with SAD with the aim of reducing the experience of loneliness among young people with social anxiety, which commonly serves to reinforce symptoms.
Rice et al. (2020b), Australia [39]	Single group, uncontrolled pre-post design	Young people with prominent social anxiety symptoms	76 (n.s., n.s.). Baseline sample: 89 (43, 48%). No power calculations are reported.	Baseline:14–25 (19.8, 13.3)	Patient Health Questionnaire-9; Male Depression Risk Scale-22; Short Warwick Edinburgh Mental Wellbeing Scale; World Values Survey—Life Satisfaction Scale; European Social Survey—Daily Activities; Abbreviated Duke Social Support Index; Revised UCLA Loneliness Scale (20-item: [79]); Social Connectedness Scale; Interpersonal Needs Questionnaire; Liebowitz Social Anxiety Scale; Brief Fear of Negative Evaluation from Others Scale; Anxiety Sensitivity Index; Social Interaction Anxiety Scale; Acceptance and Action Questionnaire; Self-Compassion Scale—Short Form; Self-Statements during Public Speaking Scale; Lubben Social Network Scale; Rosenberg Self-Esteem Scale; Emotion Regulation Questionnaire; Personal Feelings Questionnaire 2 – Brief. Pre and post.	Digital intervention ‘Entourage’ providing evidence-based therapeutic techniques for social anxiety using a social-media-based interface that allows users to build social connections, while also receiving expert clinical moderation and support from peer workers. (12 weeks)	Individual and group, tech	N/A	Sample significantly improved on clinical and social variables. Significant increases in wellbeing (*d* = 0.5), self-esteem (*d* = 0.47), and decreased loneliness (*d* = 0.63), social anxiety (*d* = 0.73) and depression (*d* = 0.66) symptoms were observed. Overall, males showed reliable improvement on 14/22 variables, whereas non-male young people reliably improved on 18/22 variables. In particular, males showed clinically significant improvement on social anxiety symptoms (*d* = 0.79), depression (*d* = 0.71), wellbeing (*d* = 0.42), and decreased loneliness (*d* = 0.46). Non-male participants experienced a significant increase in social connectedness (*d* = 0.77), reduced social anxiety (*d* = 0.78), and loneliness (*d* = 0.80).
Ştefăniţă et al. (2018), Romania [51]	In-depth interviews	Romanian Master’s students	21 (3, 14.29%)	22–30 (n.s.)	Interviews assessed the participants’ main uses of Facebook, and its influence on their subjective loneliness.	Facebook use	N/A	N/A	Only particular ways of using Facebook (e.g. reconnecting with people/staying in touch) facilitated coping mechanisms for loneliness, but unless followed by interaction or offline meetings, the advantage was reported to dissipate.
Stewart et al. (2009), Canada [56]	One-group within-subject, pilot study	Homeless (or transitioning out of homelessness) youths	14 (n.s., n.s.). Baseline sample: 70 (38, 54%)); mid-point sample 29 (n.s., n.s.). (56 participated in intervention). No power calculations are reported.	Baseline:16–24 (19, 2.5)	Social Provision Scale; Revised UCLA Loneliness Scale (20-item: [79]); Centre for Epidemiological Studies Depression Scale^f^; Proactive Coping Inventory^f^. Pre, mid (12 weeks), post.	Weekly support groups of 3–4 hours (including recreational activity and a free meal) with opportunities for one-to-one support from professional and peer mentors (20 weeks)	Group and individual, non-tech	N/A	Change in size and composition of social network was not significant; Increased satisfaction with support received was not significant; Significant decrease in loneliness but no effect size reported. Attendance at the support groups varied considerably.
	Qualitative interviews	Homeless (or transitioning out of homelessness) youths	14 (n.s., n.s.). Baseline sample: 70 (38, 54%)); mid-point sample 29 (n.s., n.s.). (56 participated in intervention)	Baseline:16–24 (19, 2.5)	Interviews Pre, mid (12 weeks), post	Weekly support groups of 3–4 h (including recreational activity and a free meal) with opportunities for one-to-one support from professional and peer mentors (20 weeks)	Group and individual, non-tech	N/A	Sample reported enhanced health behaviours, improved mental wellbeing, decreased loneliness, expanded social network, increased coping skills, enhanced self-efficacy, and diminished use of drugs and alcohol following support intervention. Sample reported emotional and informational support from mentors created a safe place to discuss problems and offered a different perspective.
Trondsen (2012), Norway [49]	Action-oriented study	Adolescents with a mentally ill parent	16 (1, 6%)	15–18 (n.s.)	Analysis of participant observation of a self-help group including messages posted on the forum (2 years).	Online self-help group: information pages, open access forum and Q&A service with health professionals (2 years)	Group, tech	N/A	Adolescents expressed intense, painful and partially overwhelming feelings such as uncertainty, fear, loneliness (e.g. lacking friends with similar experiences, not wanting to be a burden in sharing difficulties), loss and sorrow related to their parent’s mental illness. They shared strategies for handling difficult situations.
Vasileiou et al. (2019), UK [48]	Cross-sectional qualitative study	University students who had moved away from home to study, and who self-identified as experiencing loneliness	15 (6, 40%)	18–29 (21.67, n.s.)	Individual semi-structured interview. Part 1 explored how students viewed their life period at University; Part 2 examined the nature and context of the experiences of loneliness and the coping strategies participants deployed to alleviate these feelings including being asked to describe what they normally did (or avoided doing) in order to manage feelings of loneliness. (average 53 min)	None	N/A	N/A	Support-seeking, social isolation, self-reliance, problem-solving, and accommodation were the most frequent manifestations of coping with loneliness. Accommodation coping (i.e. distraction, cognitive restructuring, acceptance and minimisation) was the most commonly mentioned category of coping.

Please note that when duration of interviews or focus groups is not presented, it is absent from the paper. The number of items is included when a measure is novel to the study or is an adapted version. When (*n* = ) of sex and age is taken from baseline rather than the analytical sample, this is stated.

*n.s.* not stated.

^a^Cited as [80] but reference for 3-item version of UCLA loneliness scale is [78].

^b^“In the past 12 months, how often have you felt alone?” The response options were “Never,” “Rarely,” “Sometimes,” “Most of the time,” and “Always.” The first three options were categorised as “No.” Other responses were categorised as “Yes.”

^c^Author clarified that the 10-item version of the UCLA Loneliness Scale was a typographical error in the original paper.

^d^“I feel lonely” rated: Rarely or none of the time (less than 1 day;) Some or a little of the time (1–2 days); Occasionally or a moderate amount of time (3–4 days); Most or all of the time (5–7 days).

^e^Additional information provided by author through direct communication. Note: 12-item UCLA LS items provided but without a reference. Additional loneliness item: “How often do you feel lonely?”: Never; Seldom; Sometimes; Often; Constantly.

^f^Measures were replaced by semi-structured questions at mid and post-test.

Two unpublished M.Sc. dissertations that had been supervised by one of the authors (SJ) were included: these reported qualitative interviews with the staff of youth charities about their strategies for addressing loneliness in young people [28, 29].

#### Studies selected from previous review

Eight studies from [17] and one from [11] that met our age criterion and included mental health-related outcomes were included (Table 2).

**Table 2:** Study details for the eight included papers from Eccles and Qualter [17] and one from Ma et al. [11].

Author (year), Country	Study design	Sample	*N* in sample completing measurement at all time-points (*n* male, % male)	Age range (Mean, SD)	Measures	Intervention/ strategy and duration	Delivery method/ format	Comparison condition	Main findings
Conoley and Garber (1985), USA [12]	Randomised control trial	Undergraduate psychology students with moderate depression	57 (0, 0%). No power calculations are reported.	(n.s., n.s.)	Revised UCLA Loneliness Scale (20-item), Beck Depression Inventory, Causal Dimension Scale (controllability subscale). Pre, Post, Follow-up (2 wk) Counselor Rating Form. Post.	Reframing group: interviewer focused on ways to experience loneliness more positively; self-control group: Interviewer encouraged participants to overcome loneliness. Both groups: 2 × 30 min weekly interviews over 2 weeks.	Individual non-tech	Randomisation to one of two interventions (reframing or self-control) or control condition: wait list.	Results indicated that participants in the reframing group experienced a more significant reduction in depression than those in the self-control or control groups. All participants became less lonely over time, but no treatment was more effective than another in reducing loneliness. No differences were found for controllability. No effect sizes are reported.
Kneer et al. (2019), Netherlands [57]	Intervention study with control condition (not randomised)	Adolescent refugees	57 (22., 38.3%) 14: peers (refugees) 16: buddies (peer coaches) 12: control peers 15: control buddies. No power calculations are reported.	13–18 (14.09, 1.65)	Peers - questionnaire: leisure activities; media usage; school connectedness (3 items); school motivation (8 items); peer norms for school performance (3 items); self-esteem (10 items—1 dismissed); life satisfaction (7 items) emotional support (4 items); social distraction (3 items); affectionate support (3 items) social anxiety (9 items); peer loneliness (16 items: [81]). Buddies—semi-structured media diaries; weekly. Pre and Post.	Peer coaching ‘Peer2Peer’ to help refugee adolescents (peers) orient to new culture and build friendships. Buddies received 3 days (4 h) training. Peers met with buddy a minimum of 1 × monthly. Online communication almost daily. (14 weeks)	Individual, non-tech/ tech	Control condition: peers and buddies received no intervention/ training	Neither buddies nor peers nor. control peers showed any significant differences between pre and post. Only control buddies showed a higher school connectedness post. Social media played a central role in keeping in contact and also initiating contact, micro-coordination, and ‘hanging out’.
Larsen et al. (2019), Norway [16]	Randomised control trial	Adolescents enrolled in upper secondary schools	1937 (n.s., n.s.) 670: Intervention 1 809: Intervention 2 458: Control group (Sample at baseline 2254 (1206, 53.5%)). Power calculations reported in the study protocol [71] indicated that a sample of 975 students and 49 classes was needed to detect a small effect size of .25. It is not reported how many classes participated but authors report lack of statistical power due to low number of participating schools.	Baseline: 15–19 (16.82, n.s.)	Short form Symptom Check List (5-item); Norway Loneliness Scale (6-item: [82]); Perceived Family Wealth. Baseline and follow-up (7 mo.).	Whole-school programme ‘Dream School Program’ (DSP): aims to enhance the psychosocial environment to reduce loneliness and mental health problems. (Intervention 1 ‘single tier’); DSP + Mental Health Support Team (MST) – counsellors and nurses etc target specific students with known mental health problems/other issues (Intervention 2 ‘multi-tier’) (1 × class and poster—semester 1; 1 × class - semester 2)	Group, non-tech	Randomisation to one of two interventions (DSP or DSP + MST) or control condition: Education as usual	No effect of the intervention on students’ mental health problems and loneliness in either of the intervention groups. An overall increase in mental health problems and loneliness was found in all groups at follow-up. Compared to girls in the control group, girls in the multi-tier group had a significantly smaller increase in mental health problems. No effect size is reported.
Masia‐ Warner et al. (2005), USA [13]	Randomised control trial	Adolescents with social anxiety disorder (12% at baseline had a subtype with main concerns around performance situations and public speaking in class)	35 (9, 25.7%) 18: intervention (4, 22.2%) 17: control group (5, 29.4%). No power calculations are reported.	13–17 (14.8, 0.81) (Intervention (15, 0.59); Control (14.5 (0.94))	Independent rated: Anxiety Disorders Interview Schedule for DSM IV: Parent and Child Versions; Liebowitz Social Anxiety Scale for Children and Adolescents; Social Phobic Disorders Severity and Change Form; Children’s Global Assessment Scale. Self-rated: Social Phobia and Anxiety Inventory for Children; Social Anxiety Scale for Adolescents; Children’s Depression Inventory; Loneliness Scale (16-item: [83, 84]). Parent rated: Social Anxiety Scale for Adolescents: Parent Version Pre and post. Independent raters—pre, post, follow up (9 mo.).	Skills for Academic and Social Success ‘SASS’: Social skills 12 × 40 min weekly group, 2 × 15 min individual, 2 × group booster sessions, 4 × 90 min weekend social events; Parents—2 group meetings (90 min) (3 months).	Group/ individual, non-tech	Control condition: waitlist	Adolescents in the intervention group demonstrated significantly greater reductions than controls in social anxiety (*d* = 0.68–2.0 depending on measure), and significantly improved overall functioning (*d* = 2.3. Post - 67% intervention sample compared to 6% of waitlist sample, no longer met criteria for social phobia. No significant effect for treatment found for self-rated loneliness. Follow up (9 mo.) of intervention involved the sample only (*n* = 18) - clinical gains maintained.
Mason et al. (2016), USA [45]	Randomised control trial	Adolescents deemed to be at-risk of alcohol and marijuana use presenting at primary care clinics	117 (n.s., n.s.) 57: Intervention 60: Control group (Sample at baseline 119 (35, 29.41%): 59: Intervention (15, 25.42%); 60: Control group (20, 33.33%)) No power calculations reported.	Baseline: 14–18 (16.4, 1.2) (Intervention (16.4, 1.23); Control (16.2, 1.35))	Center for Disease Control Youth Risk Behaviour Survey—substance misuse (3 items); Social Stress Scale of the Behavior Assessment System for Children-Second Edition (BASC-2) (2-item: [85])—used to measure loneliness and perceived isolation (“I am lonely” and “People act as if they don’t hear me”, both encoded as 0 = false, 1 = true); Depression Scale of the BASC-2 (2-item). Baseline, follow-up 1 (1 mo.), follow‐up 2 (3 mo.), follow‐up 3 (6 mo.).	Substance use intervention ‘Peer Network Counselling’ (PNC) based on motivational interviewing principles to reduce social stress (1 × 20 min)	Individual, non-tech	Control condition: Participants reviewed an informational handout with a therapist covering topics related to health behaviours (e.g. exercise, nutrition/ weight management, and life skills). (1 × 20 min)	At 6 mo. follow-up PNC significantly decreased social stress (loneliness/ perceived social isolation) compared to controls, whose social stress increased (small effect size η_p_2 = 0.05). PNC temporarily moderated the effect of alcohol use, but not marijuana or heavy alcohol use.
Matthews et al. (2018), USA [53]	Randomised control trial	Adolescents with Autism Spectrum Disorder (ASD)	34 (28, 35%) 10: Intervention 1 (8, 80%) 12: Intervention 2 (10, 83.33%) 12: Control group (10, 83.33%) Authors do not report power calculations but state that “the sample size was small resulting in inadequate statistical power to detect medium and small between group differences. However, the sample size reflects the preliminary nature of a pilot study.”	13–17 (n.s.) (Intervention 1: 15.1, 1.29; Intervention 2: 15.17, 1.27; Control: 15.42, 1.08)	Parent: Social Responsiveness Scale second edition; Social Skills Improvement System; Quality of Socialisation Questionnaire – parent version. Adolescents: Test of Adolescent Social Skills; Social Interaction Anxiety Scale; Revised UCLA Loneliness Scale (20-item: [79]); Quality of Socialization Questionnaire adolescent version; Social Distance Scale; Autism Knowledge Questionnaire Pre, post, follow-up (4 mo.).	Manualised parent-assisted psychoeducational social skills intervention ‘PEERS’ teaches social and friendship skills to intellectually able adolescents with ASD using didactic lessons, role-plays and behavioural rehearsal. Parents are trained to coach their adolescents in weekly assignments. Intervention 1: traditional PEERS curriculum; Intervention 2: peer (typically developing adolescents) mediated PEERS curriculum ‘PwP’. (14 weeks).	Group, non-tech	Randomisation to one of two interventions or control condition: delayed traditional PEERS treatment condition (DTC) i.e. waiting list control.	Both interventions showed significantly greater improvements in social skills knowledge to DTC (*d* = 3.97–4.38), which was maintained at follow-up. PwP demonstrated significant increases in parent-reported social skills relative to DTC (*d* = 1.48) maintained at follow-up. Both interventions showed marginally significant improvement in loneliness (non-parametric) compared to DTC. With one extreme outlier in each treatment group excluded, significant reduction in loneliness in both intervention groups relative to DTC at post (*d* = 1.06–1.43) and maintained at follow-up.
Rohde et al. (2004), USA^a^ [44]	Randomised control trial	Incarcerated young males	109 (109, 100%) 39: Intervention 25.: control group 1 45: control group 2 (Sample at baseline 138) Authors report that “with α set at .05, two-tailed, we had adequate power (>0.80) to detect medium effect sizes or larger (Cohen f > 0.33) for intervention main effects.”	Baseline: 12–22 (n.s.) (intervention and control group 1: (16.3, 1.9); control group 2: (16.8, 1.7))	Youth Self-Report; Current Suicidal ideation and Lifetime Suicidal Attempts; Life Attitudes Schedule - Short Form (24-item); Coping Skills (15-item); Rosenberg Self-Esteem Scale; UCLA Loneliness Scale (8-item: [86]); Subjective Probability Questionnaire (5-item); Social Adjustment Scale-Self Report for Youth (4-item) Knowledge of CBT Concepts (10 short answer items). Pre and post.	CBT Coping Course (modified version of the Adolescent Coping with Depression Course, Clarke et al. (1990)) Biweekly meetings, 16 × 90 min sessions over 8 weeks	Group, non-tech	Control condition: usual care	Significant improvement in treatment group compared to controls at post-treatment in seven of the 17 examined measures: Youth Self-Report externalising scores (*η*^2^ = 0.07), three measures from the Life Attitudes Scale (total score *η*^2^ = 0.11, death-related *η*^2^ = 0.09, self-related *η*^2^ = 0.11), self-esteem (*η*^2^ = 0.10), one measure of social adjustment (*η*^2^ = 0.07), and CBT knowledge (*η*^2^ = 0.13). All reported effect sizes are small. No significant impact was found on any of the other measures including impact on loneliness.
Smith et al. (2017), Canada [43]	Single-group intervention study	Gay and bisexual young men	19 (19,100%) (Sample at baseline = 33 (33, 100%)). No power calculations are reported but the stated aim of the study was to assess feasibility and provide preliminary evidence of its efficacy so this should be considered a pilot study.	Baseline: 18–25 (21.91, 2.15)	Center for Epidemiological Studies – Depression; State-Trait Inventory for Cognitive and Somatic Anxiety—state version; UCLA Loneliness Scale (20-item: [80]); Rosenberg Self-Esteem Scale; Nungesser Homosexuality Attitudes Inventory Revised; Sexual Orientation Concealment Scale; Lesbian, Gay, and Bisexual Identity Scale; Condomless sex and number of sex partners (4 categories); Alcohol and drug use frequency (8 substances). Pre, post, follow-up (3mo).	Small group intervention aimed at reducing negative mental and behavioural health outcomes resulting from minority stress. ‘Project PRIDE’ (Promoting Resilience In Discriminatory Environments). Bi-weekly sessions 8 × 2.5 h (4 weeks)	Group, non-tech	N/A	Comparing pre- to post-treatment: small, significant effect sizes for reduced loneliness (*d* = −0.36 post, -0.35 follow-up), increased self-esteem (*d* = 0.27 post, 0.88 at follow-up, reduced internalised homonegativity *d* = −0.21 post, −0.28 follow-up, and reduced difficult process related to sexual orientation identity (*d* = −0.30 post, −0.29 follow-up. Most changes remained stable or increased to follow-up; self-esteem continued to significantly increase.
Stice et al. (2010), USA^b^ [42]	Randomised control trial	At‐risk adolescents with elevated depressive symptoms	341 (150, 44%) 89: Intervention 1 88: Intervention 2 80: Intervention 3 84: Control group. No power calculations reported.	14–19 (15.6, 1.2)	Beck Depression Inventory; Automatic Thoughts Questionnaire; Pleasant Events Schedule; Emotional Expression (9-item); UCLA Loneliness Scale (8-item: [86]). Pre and post.	Cognitive Behavioural (CB) Group (Intervention 1) and Supportive Expressive (SE) Intervention Group (Intervention 2)—6 ×1 h weekly sessions. Bibliotherapy (Intervention 3) ‘Feeling Good’ by Burns, D. (1980). Controls given NIMH brochure describing major depression and recommends treatment for depressed youth – ‘Let’s Talk About Depression’, NIH and information about treatment options.	Group (interventions 1 and 2), non-tech; Individual (intervention 3), non-tech	Randomisation to one of two intervention group conditions, individual bibliotherapy, or control condition: assessment and brochure	Compared to controls both the CB (*r*_p_ = −0.32) and SE (*r*_p_ = −0.17) groups showed significant reductions in depressive symptoms, whereas there was no significant difference for the Bibliographic group. Only the CB group showed a significantly greater decrease in loneliness relative to controls (no effect size reported). CB also showed a significant decrease in negative cognitions (*r*_p_ = −0.3) and increase in pleasant activities (*r*_p_ = 21) compared to controls. Compared to controls the SE group showed no significant difference in change in loneliness but did show a significant difference in change in emotional expression (*r*_p_ = −0.3). Change in loneliness (*r*_p_ = −0.32) but not emotional expression, predicted change in depressive symptoms for the SE condition.

Number of items is included when a measure is novel to the study or is an adapted version. When the number of sex and age is taken from baseline rather than the sample completing the study, this is stated.

*n.s.* not stated, *d* = Cohen’s *d*, *η*_p_^2^ = partial eta squared, *r*_p_ partial regression coefficient.

^a^Number in intervention group and control group 1 not included in paper and established via personal communication with the author.

^b^Some information included in this table is taken from an earlier study by ref. [87].

#### Grey literature

Twenty-five sources from UK-based third sector organisations or public bodies related to addressing youth loneliness and mental health were included (Table 3). Although some sources included younger age groups, only information relating to 14–24-year olds was used in developing the framework.

**Table 3:** Included third sector and policy evidence from 25 sources (reports, websites, projects and resources).

Charity or Organisation	Title (Date of publication) Link to website	Description of source and age of children	Findings or recommendations
Reports			
Office for National Statistics [68]	Children’s and young people’s experiences of loneliness. (December 2018) https://www.ons.gov.uk/peoplepopulationandcommunity/wellbeing/articles/childrensandyoungpeoplesexperiencesofloneliness/2018	Analysis of children’s and young people’s views, experiences and suggestions to overcome loneliness using interviews and surveys. 10–24 years old	Suggestions to tackle loneliness: —Reaching out to others for emotional support and advice, participating in activities, clubs and sports, going to community spaces where you might meet new people, and volunteering —Putting loneliness on the school curriculum, preparing young people for life transitions and what to expect, increasing and augmenting support from pastoral care managers or counsellors —Societal approaches to change the way we deal with loneliness and to create a culture of openness e.g. talking about loneliness more openly as we do with mental health.
The Children’s Society [62]	Loneliness in childhood. Exploring loneliness and well-being among 10–17 year olds. (March 2019) https://www.childrenssociety.org.uk/sites/default/files/loneliness.in_.childhood.2019-compressed.pdf	Children completed the household survey in May and June of 2018. The survey covers 2,000 households in England, Scotland and Wales, and is socio-economically representative. 10–17 years old	This report identifies the importance of strong relationships in tackling loneliness. This includes family relationships, relationship with friends, and relationship with other adult role models. Community building, tackling bullying and access to more specialised mental health support are also touched upon.
Co-op Foundation [50]	We are lonely, but not alone. How young people are beating loneliness, and what we can all do to help. (September 2019) https://www.coopfoundation.org.uk/wp-content/uploads/We-are-lonely-but-not-alone-Research.pdf	Report considering how young people are responding to loneliness. 10–25 years old	Findings: —Most young people show a self-help spirit when it comes to tackling loneliness —Those who had felt lonely used on average two to three different techniques to address this —The self-help approach has limitations; there was a mis-match between the approaches most widely tried by young people and those which were most likely to be effective —The loneliest young people, who may lack existing support networks from family or friends, face particular challenges —The majority of young people would be comfortable helping others their age who may be lonely —Peer support remains an under-used resource —Perceptions that youth loneliness is not taken seriously by society.
Department for Digital, Culture, Media and Sport and Office for Civil Society [88]	Loneliness Annual Report. (January 2020) https://www.gov.uk/government/publications/loneliness-annual-report-the-first-year/loneliness-annual-report-january-2020--2	Update on governmental loneliness strategy. All ages	Young people struggle with loneliness more than any other group, but targeted interventions and policies are currently relatively sparse. Report focuses on the need for further policies targeted at tackling children and young people’s loneliness; the need for more information and communication about loneliness and the activities which are available to reduce it; and the need to tackle loneliness through place (strengthening community infrastructure and assets, and growing people’s sense of belonging). Strategic commitments include primary and secondary school children being taught about loneliness from September 2020.
Barnardo’s [89]	Left to their own devices: Young people, social media and mental health. (June 2019) https://www.barnardos.org.uk/sites/default/files/uploads/B51140%2020886_Social%20media_Report_Final_Lo%20Res.pdf	The main aim of this report is to understand the views of children and young people regarding the impact of social media on mental health and wellbeing. The report also discusses the effect of social media in relation to isolation and loneliness. Under-5 to 19 years old	Findings: —Social media can be beneficial in reducing isolation and loneliness among children and young people. —Through creating and maintaining real world connections online, children and young people can expand their ‘social capital’ and therefore reduce loneliness. —Social media can play a role in helping children and young people who, as a result of illness, may not have the opportunity to physically meet with others.
Mental Health Foundation [67]	State of a generation: Preventing mental health problems in children and young people. (November 2019) https://www.mentalhealth.org.uk/publications/state-generation-preventing-mental-health-problems-children-and-young-people	This report discusses loneliness in the context of mental health. Children and young people up to 25 years old	It finds that many young people: —Are unable to speak about their emotions with others —Feel isolated and lonely —Lack companionship —Lack a trusted adult to go to for advice and support if they are experiencing a problem (including mental health problems) Also discusses the feelings of fear, isolation and loneliness caused by the stigma, discrimination and abuse experienced by people with learning disabilities.
Samaritans [69]	Loneliness, suicide and young people. (January 2019) https://media.samaritans.org/documents/loneliness-suicide-young-people-jan-2019.pdf	Report outlining findings from a literature review, an online survey with 250 young people who had felt lonely and suicidal at some point(s) in their lives, interviews with 15 young people on their experiences of loneliness and suicidal thoughts, and a roundtable with policy experts. 16–24 years old	Recommendations include to: —Include loneliness in training for practitioners who work with young people, specifically those who are at risk, to improve the number of young people who are identified for early help and support —Roll out national awareness campaigns to tackle the stigma that many young people are experiencing around loneliness —Take a public health approach when commissioning services for young people, e.g. social prescribing.
Sense [61]	“Someone cares if I’m not there”: Addressing loneliness in disabled people. (October 2017) https://www.nat.org.uk/sites/default/files/publications/loneliness_report_-_someone_cares_if_im_not_there.pdf	A report by the disability charity Sense for the Jo Cox Commission on loneliness, on behalf of a coalition of disability charities to collectively highlight the issue of loneliness for disabled people. All ages including children and young people	Recommendations: —Increasing awareness, improving social attitudes - Enabling independence through access to social care —Tackling poor accessibility —Providing fair and adequate financial support —Increasing access to employment and work experience.
Action for Children [63]	It starts with hello. (November 2017) https://www.actionforchildren.org.uk/media/9724/action_for_children_it_starts_with_hello_report__november_2017_lowres.pdf	A report looking into the impact of loneliness in children, young people and families. Includes a parental survey. Children and young people of all ages including 11–25 years old	Examples of current schemes which provide support to develop social skills, promote resilience and reduce isolation include: —Anti-bullying programmes in schools and colleges, youth clubs, sports clubs and online —Personal, Social and Health Education (PSHE) at all levels of schooling, including Life Skills for older children —Mental health support on site in all educational settings —Peer counselling, local befriending or mentoring services —Opportunities to help others through volunteering or working in the community.
UK Youth [90]	A place to belong: The role of local youth organisations in addressing youth loneliness. (August 2018) https://ukyouth.org/wp-content/uploads/2018/08/A-Place-To-Belong-The-role-of-local-youth-organisations-in-addressing-youth-loneliness.pdf	This report focuses on the role of local youth organisations in addressing youth loneliness from the perspective of youth workers. (Includes a quantitative online survey focus groups, and in-depth interviews.) 9–25 years old	Recommendations: —Support for further research and consultation with youth workers, young people and experts to develop a youth sector-wide strategy for youth loneliness —An increase in core funding to enable existing local youth organisations to provide support to young people at risk of loneliness —An increase in funding for detached work to allow youth organisations to better engage those young people who can’t access, or aren’t accessing, youth services. —Development of tools and resources to help youth workers raise awareness of loneliness, appropriately and effectively among all young people —Development of activities and resources to help youth workers identify young people at risk of loneliness, and young people to identify themselves and their peers as at risk and in need of support —Development of activities and funded programmes that build resilience and strong support networks—two key protective factors in reducing the risk of loneliness.
ACEVO [91]	Coming in from the Cold: Why we need to talk about loneliness among our young people. (May 2020) https://www.acevo.org.uk/wp-content/uploads/2019/07/Coming-in-from-the-Cold.pdf	Report on loneliness among young people in London emphasising the cost benefits of reducing loneliness in this age group. Approximately 16–26 years old	Recommendations: —Building personal resilience and capacity to form healthy relationships. —Building communities.
Websites			
Young Women’s Trust [92]	‘Lifetime of loneliness: one in four young people feels lonely, finds Young Women’s Trust’. (January 2019) https://www.youngwomenstrust.org/what_we_do/media_centre/press_releases/904_lifetime_of_loneliness_one_in_four_young_people_feels_lonely	Webpage reporting the survey carried out for Young Women’s Trust (a charity that supports young women on low or no pay). 18–30 years old	A lack of close relationships is a possible reason for loneliness. Feeling isolated impacts on young women’s confidence and their mental health. Combined with a lack of networks, this can make it harder to look for jobs and can lead to young women being shut out of the labour market. More support is needed for young women who want to work. This includes mentoring to help ease women’s move back into education or employment. The charity argues that tackling loneliness would benefit individuals, businesses and the economy.
Mind [93]	Tips to manage loneliness. (July 2019) https://www.mind.org.uk/information-support/tips-for-everyday-living/loneliness/about-loneliness/	Mental health charity website. Explains loneliness, including the causes of loneliness and how it relates to mental health problems. Gives practical tips to help manage feelings of loneliness, and other places you can go for support. All ages	Tips and suggestions for managing feelings of loneliness: —Take it slow —Make new connections —Try peer support —Try to open up —Talking therapies —Social care —Be careful when comparing yourself to others —Look after yourself.
Childline [94]	Loneliness and isolation. (no date available) https://www.childline.org.uk/info-advice/your-feelings/feelings-emotions/loneliness-isolation/	Website of children’s counselling service (online and telephone) run by the National Society for the Prevention of Cruelty to Children (a child protection charity). Children under 19 years old	The website provides a range of recommendations including a ‘guide for developing trust’ and suggestions for ‘things that can help if you feel lonely’. These include support from other people on Childline’s message boards, tracking feelings on their mood journal, using their Art box to draw or write down thoughts, and talking to a Childline counsellor for support.
The Source [95]	Feeling lonely. (no date available) https://www.thesource.me.uk/health/feeling-lonely/	Local website with information and advice for young people in Suffolk. Children and young people of all ages	Recommendations: —Re-connect with people around you —Tell someone how you are feeling (if you don’t who to talk to, you could text a school nurse on for free confidential advice and support) —Remember you are loved and valued for being amazing you, even if you don’t feel like it sometimes —Get involved in something e.g. volunteering —Find a local youth group —Search for leisure activities and groups in your area —Emotional wellbeing hub for children and young people’s —The website also gives practical advice if you are nervous about joining a new group/activity for the first time.
YMCA [59]	Ending the loneliness epidemic amongst young disabled people. (August 2017) https://www.ymca.co.uk/youth-opportunity/news/ending-loneliness-epidemic-amongst-young-disabled-peopled	Webpage of local central London YMCA describing efforts to provide accessible social spaces. Young people especially 16–19-year-olds	A lack of understanding about disabilities leads people to avoid those with them, and consequently makes it harder for disabled people to make and maintain lasting friendships. Often people without a disability don’t believe they have anything in common with those who do. There are also numerous practical barriers to social connections that disabled people face, such as inaccessible facilities, transport links and inappropriate social care. The club currently runs an inclusive sports club every Sunday, works with local schools to put on yoga classes for those with special educational needs and disabilities, is currently running a week-long cooking course for those with disabilities. YMCA Training also delivered apprenticeships to 144 learners with a disability.
Resources			
Educare [96]	Supporting children and young people with loneliness. (no date available) https://www.educare.co.uk/Media/Supporting%20children%20and%20young%20people%20with%20loneliness.pdf	Resource for parents created by Educare (part of TES; a weekly UK publication aimed at education professionals). Children of all ages	Recommendations: —Having someone to talk to —Strengthening family relationships —Strong relationships e.g. peer relationships and friendships —Positive adult role models —Finding opportunities for children and young people to spend time with caring and inspiring adults —Spending time outside or with animals —Ensuring a good diet, staying hydrated, getting enough sleep —Online support: to reach out to others with similar interests, share experiences and ask advice.
Public Health England and UCL Institute of Health Equity [64]	Local action on health inequalities: Reducing social isolation across the life course. (September 2015) https://assets.publishing.service.gov.uk/government/uploads/system/uploads/attachment_data/file/461120/3a_Social_isolation-Full-revised.pdf	Practice resource which emphasises that social isolation and the relationship with health and inequalities in health is complex and multi-factorial. All ages including children and young people	Recommendations and findings: —Support children and families in building good quality relationships —Tackling bullying by families, schools and the wider community to generate positive and inclusive attitudes to all —Young carers are a group at increased risk of social isolation whose needs are unlikely to be met —The potential of the built environment to impact on social isolation.
Ambitious about Autism [60]	Include Autism. (April 2019) https://www.ambitiousaboutautism.org.uk/what-we-do/youth-participation/youth-led-toolkits/include-autism	This resource has been created by members of the charity’s Youth Council to help tackle the problem of loneliness and social isolation felt by many of their peers by helping more autistic young people access youth groups and after school activities.	It is designed give those running youth groups or clubs a better understanding of autism and how to support autistic young people. The toolkit offers advice on what autism is and how to talk about it positively. Tips include: —Giving new members the option of visiting the group before they start —Dim the lightbulbs, use natural light, or allow sunglasses —Say a person’s name before giving clear and straightforward instructions —Allow headphones or ear-defenders and have a designated quiet room —Have a visual agenda showing what is going to happen —Offer new members a buddy to show them where to go and what to do.
Projects			
Manchester Metropolitan University, 42nd Street (funded by the Co-op Foundation) [97]	Loneliness Connects Us. (2016–2019) http://www.lonelinessconnectsus.org/	Project looked at the use of artistic and creative methods to both explore and create strategies to reduce youth loneliness. 42nd Street is a local charity that supports young people with your emotional wellbeing and mental health. 11–25 years old	Recommendations and findings: —Develop new ways of thinking and talking about youth loneliness —Restore threatened youth work provision so that all young people have someone who knows and accepts them for who they are —Interventions should go beyond individual funded projects and towards commons spaces and social movements to bring into being more co-operative and convivial communities —Arts-based and creative methods create spaces and relationships where young people can find connection and navigate painful forms of loneliness.
Young Minds [98]	Using photography to tackle loneliness. (November 2018) https://youngminds.org.uk/blog/using-photography-to-tackle-loneliness/	Young Minds is a child and young people’s mental health charity. Capture Change was a project for young people from Southwark. Child or young person up to 25 years old	The Capture Change project used photography to explore what loneliness and belonging meant to young people, and developed participant’s skills and confidence to raise awareness about these topics amongst their peers. Each participant was given a camera for the duration of the project and was asked to respond to a series of questions through photography. Each day of the project, participants prepared small fieldtrips in the neighbourhood to spaces they felt could create a sense of belonging and connection for young people.
Co-op Foundation and NCP [99]	The Building Connections Fund: Co-design and community spaces. (June 2019) https://www.thinknpc.org/wp-content/uploads/2019/08/BCF-CDCS-Final-Report-updated.pdf	A qualitative evaluation of 144 government-funded projects to maximise the use of community spaces for young people. This is to better understand the role of co-design and community spaces in reducing loneliness for young people. 8–25 years old	Recommendations and findings: —Organisations showed little explicit focus on the potential of community spaces to reduce loneliness —For many young people, their experience of loneliness or isolation may be most painfully felt in the online space —Co-design was found to improve how participants view loneliness, and how they talk about it with others. It helped participants build friendships and improve their social skills. It increased their confidence, which can unlock other skills and interests —Created a safe space for people to talk about loneliness, critical for enabling and facilitating conversations.
MindOut [100]	Peer Support Groups. (no date available) https://www.mindout.org.uk/get-support/peer-support-groups/	MindOut is a mental health service that works to improve the mental health and wellbeing of LGBTQ communities and to make mental health a community concern. Under 30 years old	The peer support groups are confidential, non-judgemental, independent from other services, free of charge and run by experienced mental health workers. There is a specific Under-30’s group for young people. This provides opportunities for LGBTQ people to: —Meet others with shared lived experiences and identities —Create a safe and supportive space —Help reduce loneliness and isolation and share coping strategies.
Anna Freud Centre [101]	Help Create a New Wellbeing App for Young People! (no date available) https://www.annafreud.org/on-my-mind/get-involved/research-and-studies/ketka/	Call for young people to help in the creation of wellbeing app. 14 to 19 years old	To combat the intensified feelings of loneliness and isolation, an online platform is being developed for young people to positively connect with each other throughout this uncertain time. They are looking for young people to participate in a virtual workshop to co-design the new platform.
Co-op Foundation and Effervescent [70]	Lonely Not Alone. (September 2019) https://www.coopfoundation.org.uk/news/lonely-not-alone-campaign/	The campaign was created by a group of nine young people who’ve felt lonely in the past, to beat the stigma of youth loneliness, in partnership with the specialist youth co-design agency Effervescent.	‘Lonely Not Alone’ campaign encourages everyone to wear yellow socks to show they care about youth loneliness. Supporters can then post a picture of themselves online using #LonelyNotAlone. People are invited to get involved whenever and wherever they like. It is hoped that every time someone wears yellow socks, they will show young people everywhere that youth loneliness matters.

### Quality assessment

Although the CIS approach traditionally does not include quality assessment but instead focuses on the relevance of studies in order to build a conceptual map of the topic in question [18], we provide quality ratings of all the included studies in Supplementary Table S2, as this may be helpful in considering possible further research and clinical potential of the approaches discussed. Consistent with [14], a shortened quality assessment using criteria adapted from the National Institutes for Health (NIH) was used to assess the quality of included quantitative studies and final rating are given as ‘poor’, ‘fair’ and ‘good’. The Consolidated Criteria for Reporting Qualitative Research [COREQ: 30] rating scale was used to assess the quality of included qualitative studies, and for these raw scores are given as a proportion of the number of relevant rating items, as the COREQ does not provide guidance on how to convert raw scores to a categorical rating of quality. In the main text we discuss the quality ratings for the included studies only in relation to those that used randomised controlled methods: we discuss study quality in relation to RCTs in order to provide context on how confident we can likely be about the outcome findings for these studies, and we focus on RCTs because it is only this study design that can provide meaningful information on effectiveness.

### Coding

**Fig. 2:**
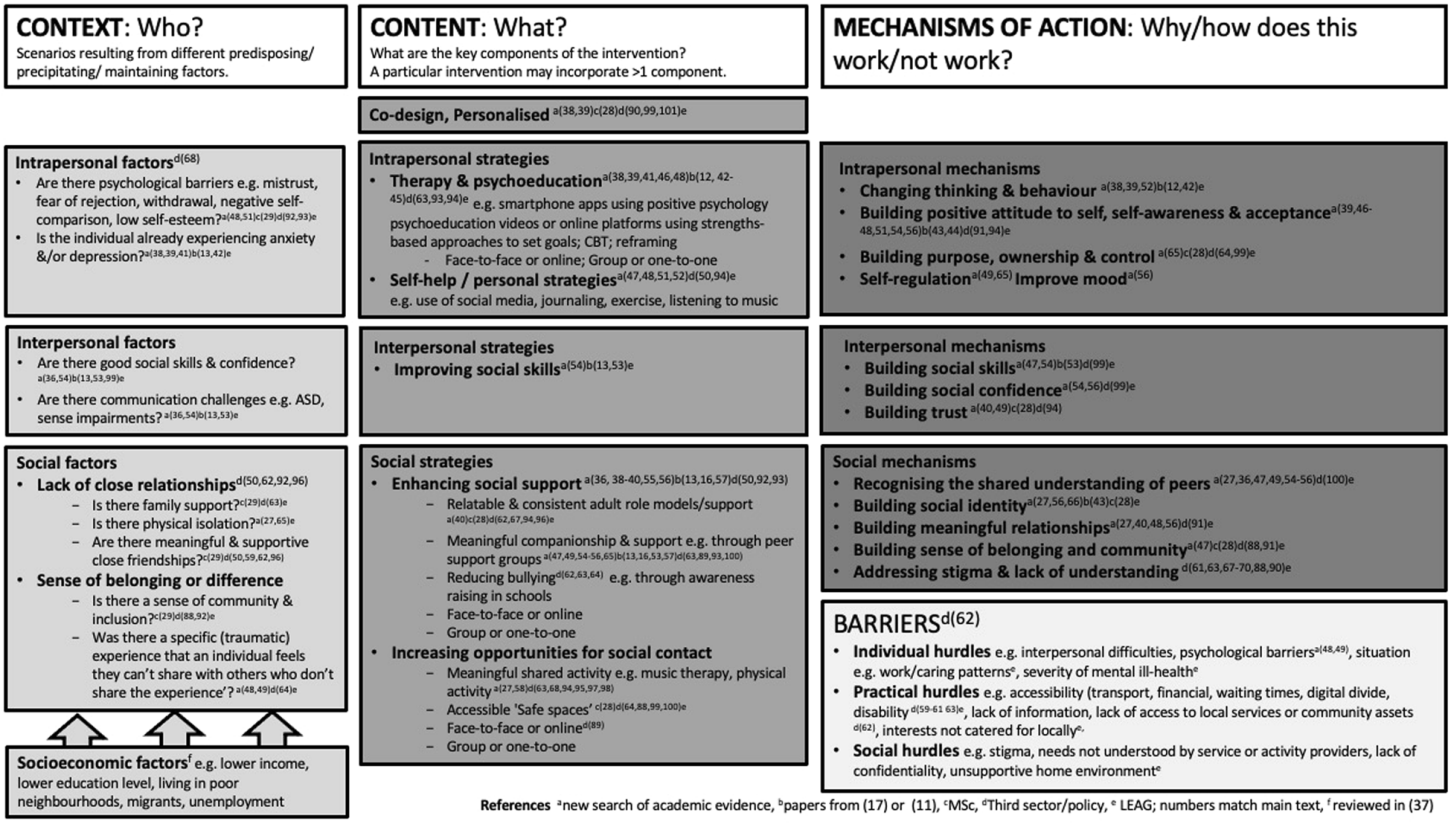
Conceptual framework of interventions to address loneliness in young people. Synthetic constructs are given in bold and are structured under the overarching themes of Context (who an intervention might work for), Content (what the intervention involves), Mechanisms (how and why an intervention might work) and Barriers (why an intervention might not work). Sub-constructs are bullet-pointed and given in bold, and their attributes are also provided. References: anew search of academic evidence, ^b^papers from [17] or [11], ^c^MSc, ^d^Third sector/policy, ^e^LEAG; reference numbers match the main text, ^f^reviewed in [37].

Where interventions related to more than one type of strategy (e.g. intrapersonal therapy and peer support), these were coded within the relevant category for the dominant approach. For example, the online Entourage platform delivers evidence-based therapeutic content to address social anxiety, and is coded and discussed as an individual-level therapeutic intervention, even though it also included a peer support element. However, interventions that used multiple approaches are referenced in all relevant categories in Fig. 2.

### Synthesis

Through CIS, the underlying data are transformed into ‘synthetic constructs’: higher-order theoretical concepts that capture diverse evidence [18, 25, 31]. These concepts summarise the key overarching themes in a diverse body of evidence, which may not be found in the literature being synthesised itself. Identifying these constructs requires questioning underlying assumptions in the literature, and thus offering a critical interpretation of the evidence. In the CIS approach, each ‘synthetic construct’ has ‘attributes’, which are the characteristics that define it and can be thought of in a similar way to subthemes in qualitative analysis.

To build a coherent framework, we drew on elements of a conceptual model developed through CIS [31], pertaining to ‘Context’ (population characteristics and setting), ‘Content’ (what the key elements of strategies are, where ‘strategy’ encompasses both formal interventions and broader coping strategies), and the proposed ‘Mechanisms of Action’ that mediate effectiveness based on individual context (Fig. 2). In addition, we synthesised the construct ‘Barriers’. To incorporate stakeholder input following RRR principles, we extracted themes from notes taken during initial discussions with the LEAG and these were used in coding the academic and grey literature, with additional themes being added as necessary. Using the themes raised during consultation with the LEAG allowed us to bring a critical perspective to the academic literature to identify key gaps in existing strategies (e.g. addressing family relationships and stigma) and to understanding possible mechanisms of action as well as potential barriers, which were not always clear in the intervention studies. Critically comparing the academic literature with grey literature from the third sector also highlighted gaps in the evidence base (e.g. place-based approaches). By drawing together stakeholder input (an innovation to the CIS method based on RRR), quantitative and qualitative academic literature and evidence from third sector and policy grey literature, we iteratively synthesised cross-cutting ‘Intrapersonal’, ‘Interpersonal’, and ‘Social’ constructs, in addition to nested sub-constructs (Fig. 2). These constructs identified the key elements of different types of strategies to address loneliness and their potential mechanisms of action.

## Results

Twenty-seven studies (total participants *n* = 105,649; range 1–102,072) were included (Fig. 1): 18 from the new searches (Table 1), eight from [17] and one from [11] (Table 2). Twenty-five third sector sources (Table 3) and two M.Sc. dissertations [28, 29] were also included. Please see Supplementary Materials for discussions of the loneliness (Supplementary Tables 3−5), and anxiety and depression measures used in the included studies.

We first outline a conceptual framework of potentially promising approaches for different needs, and the possible mechanisms by which these might work. Iterative development of the framework involved discussions of the review results with academic and lived experience experts and yielded a visual summary of interventions for potential future development and testing, and their content, mechanisms and potential applications (Fig. 2). Within this framework, we then discuss outcomes and study quality for the randomised controlled studies only, as other study designs do not meaningfully pertain to assessing effectiveness. Outcomes for all studies (including effect sizes where available) are summarised in Tables 1 and 2.

We started by categorising the Content as ‘Intrapersonal’, ‘Interpersonal’ and ‘Social’ and then identified the contextual factors that might lend themselves to that particular strategy, and the mechanisms by which the strategy might work, to create three ‘pathways’. ‘Intrapersonal’ level constructs are taken to be those that relate to psychological characteristics and mechanisms and the strategies that specifically target these internal characteristics and mechanisms, including steps that an individual has taken themselves to manage their internal psychological states, such as journaling or exercising to moderate their mood. We use ‘Interpersonal’ to refer to individual-level factors, strategies and mechanisms that require interaction with others: the behavioural manifestations of ‘Intrapersonal’ psychological factors. Although Interpersonal factors are also inherently social, in this framework we use ‘Social’ to refer to strategies that target social interaction per se rather than the underlying psychological (e.g. trust) and behavioural (e.g. social skills) elements involved in a social interaction. ‘Social’ factors and mechanisms of action are taken to be those that relate to the presence or absence of satisfying intimate and community relationships. We acknowledge that there is overlap between these categories: for example, self-confidence and social skills are individual-level variables but we have focused on their behavioural manifestations and therefore describe these as interpersonal-level characteristics. Similarly, although having ASD is an intrapersonal characteristic, the social difficulties that people with ASD encounter are often to a considerable degree the result of negative societal attitudes and expectations that they will impersonate ‘neurotypical’ behaviour. We therefore categorise the communication challenges and difficulties with ‘neurotypical’ social skills encountered by people with ASD as key contextual factors with regard to loneliness, listing them as ‘Interpersonal’ factors, rather than Intrapersonal ones. Moreover, ‘recognising the shared understanding of peers’ is a psychological change in thinking but has been listed under'Social’ mechanisms because it appears to be a key mechanism of change for ‘Social’ strategies that enhance social support or increase opportunities for social contact, and has thus been included in the ‘Social’ pathway. ‘Sense of belonging’ could similarly be listed as an Intrapersonal factor, but has been listed under ‘Social’ because it is the key contextual factor for strategies that increase opportunities for social contact.

### Context

‘Context’ captures variation in possible factors underlying an individual’s loneliness. Consequently, ‘Context’ affects which strategy might be feasible, acceptable and effective for particular individuals. Rather than focusing on specific demographic groups, the synthetic constructs within ‘Context’ represent key causes of loneliness that could result from different combinations of predisposing, precipitating and maintaining factors (Fig. 2). For example, a young person may be hospitalised, a refugee, or have recently started university, but all of these experiences could lead to ‘Social Factors: Lack of Close Relationships’. These constructs were drawn out of consultation with the LEAG about possible underlying causes of youth loneliness and formulated with reference to the included published and grey evidence, as well as conceptualisations of loneliness from the broader literature (e.g. the distinction between emotional and social loneliness [32]). A combination of these factors might precipitate or maintain an individual’s loneliness.

‘Intrapersonal Factors’ include whether anxiety and depression are already present, and psychological barriers associated with loneliness, such as cognitive biases [33], low interpersonal trust [34], and low self-esteem [35]. ‘Interpersonal Factors’ primarily relate to specific groups facing challenges with communication skills, such as those with ASD, or who lack social confidence, such as those with social anxiety, but might be more broadly applicable (e.g. [36] found that lonely university students reported they felt they lacked social skills). ‘Social Factors’ relate both to lacking or unsatisfactory close emotional relationships with family and friends (‘emotional’ loneliness) and lacking a wider sense of community belonging (‘social’ loneliness), since loneliness can be experienced in relation to one or both of these [32].

These proximate individual-level factors are seen against the backdrop of wider ‘Socio-economic Factors’. For instance, although Lim et al.’s [37] recent review and proposed model of loneliness across the life-course reported limited evidence for the impact of socioeconomic status, greater loneliness was found to be associated with lower income, lower educational attainment, having more economic problems, living in poor neighbourhoods and being a migrant. Such factors can create both loneliness and barriers to addressing loneliness (see ‘Barriers’). These Socioeconomic Factors may lead to loneliness via Intrapersonal, Interpersonal and Social Factors and we focus on these potentially mediating factors in this review, due to the need to develop individual-level clinical and social intervention strategies. It is beyond the scope of this current review to address potential socio-political strategies to address socioeconomic inequalities and thus loneliness, but such strategies are likely to play a major role in reducing loneliness and preventing and alleviating anxiety and depression in this age group (and beyond), and research in this area is much needed.

### Content

The ‘Content’ (sub)constructs outline six key active ingredients of strategies to reduce loneliness in young people (Fig. 2).

### Content: co-designed and personalised

The ‘Co-designed and Personalised’ construct highlights both that young people need to be integrally involved in the development and testing of intervention strategies, and that different strategies may work for different individuals, and for the same individual at different times. Co-designed and personalised interventions may be individual or collective, and the key element is that strategies suit each individual and their needs, for example, through a flexible modular approach that might combine individual, dyadic and collective elements. The LEAG highlighted the importance of engaging young people in developing strategies to reduce loneliness and the need to address individual needs and interests. The ability to modify intervention delivery may be a key component of success. For instance, the online platform Entourage uses a participant’s unique strengths profile to personalise therapeutic suggestions for social anxiety, and piloting suggests it has potential for reducing loneliness [38, 39]. Different strategies may be needed for different individuals, and over time for the same individual. For example, the LEAG suggested that therapeutic input to manage psychological barriers may subsequently allow better engagement with community-based social opportunities later on. Equally, enhancing meaningful social support may facilitate effective therapeutic processes [40]. Consequently, effective interventions may require multiple elements, depending on individual ‘Context’.

### Content: intrapersonal strategies

In contrast to previous loneliness intervention taxonomies, we do not use the terms ‘changing cognitions’ [22] or ‘addressing maladaptive social cognition’ [21] for psychological interventions, in order to encompass a broader range of Intrapersonal Strategies that also included psychoeducation and mood regulation. Eight quantitative studies used some form of ‘Therapy’ (Tables 1 and 2). Two interventions for social anxiety involved online or smartphone platforms using positive psychology content designed to improve relationship quality and facilitate social goals [39, 41]. Another study looked at cognitive behavioural therapy (CBT) for high school students reporting depressive symptoms and the mediating effect of loneliness [42], and one looked at the effect of reframing in female college students experiencing loneliness and depression [12]. Studies looking at young people not explicitly experiencing mental ill-health used in-person interventions and focused on groups potentially at risk of loneliness, such as gay and bisexual [43] or incarcerated [44] young men, adolescents at risk of substance abuse presenting at primary care clinics [45], or ‘runaway’ adolescent girls [46].

‘Self-help or Personal Strategies’ could include both direct forms, such as therapeutic apps [41] or self-reflection [47], and indirect forms, such as exercise or listening to music [48, 49]. However, the Co-op Foundation [50] reported a mis-match between the self-help approaches most widely tried by young people, and subjective reports of what helps. For example, ‘waiting for the feeling to pass’ was not always helpful, and ‘trying to make new friends’ seemed a less reliable way of addressing loneliness than turning to existing friends and family. Young people reported that social media can exacerbate loneliness, for example because a contact failed to respond or connections felt inauthentic [51]. One intervention involved quitting social media [52].

### Content: interpersonal strategies

Following Masi et al.’s [21] taxonomy of loneliness interventions, the key Interpersonal Strategy is ‘Improving social skills’. Two interventions for using this approach were delivered to people with ASD [53, 54]; in one of these, social skills training was part of an intervention specifically for university students [54]. It is worth noting that interventions aimed at improving social skills for individuals with ASD have been criticised for promoting ‘neurotypical’ social skills, and that LEAG members identifying as having ASD preferred the term ‘communication challenges’ and emphasised that people with ASD may have different ways of interacting that are not necessarily problematic. The LEAG suggested that social spaces that allowed individuals with ASD to engage socially without having to ‘camouflage’ by adopting ‘neurotypical’ social skills would be highly beneficial. A third, school-based, social skills training intervention was designed to help adolescents with social anxiety [13].

### Content: social strategies

Following Masi et al.’s [21] taxonomy of loneliness interventions, the key Social Strategies are labelled ‘Enhanced Social Support’ and ‘Increasing Opportunities for Social Contact’. Interventions that ‘Enhanced Social Support’ appeared feasible and acceptable. Approaches included an online peer support forum for university students [36], a Moderated Anonymous Online Group (MAOG) for young adults not in employment or education [55], an in-person school-based intervention comparing peer mentorship versus both peer mentors and a staff mental health support team [16], and in-person peer support groups for homeless youth [56]. A one-to-one peer support intervention for refugee adolescents involved both in-person and online communication [57]. In terms of strategies for helping those already experiencing mental ill-health, a case study reported that meaningful close relationships allowed a young woman to engage more fully with therapy for post-traumatic stress disorder [40].

Meaningful shared activities provided ‘Increasing Opportunities for Social Contact’, as illustrated by the impact of music therapy on hospitalised young people:

“…I don’t feel lonely anymore cause I’m surrounded by people who are all talking or sharing one common thought like what beat are we doing or what is going to come next….” ([27]: page 59)

Music therapy not only brought participants together, but also created a new activity to share with family [27]. Equally, engaging with physical education classes and active leisure time was found to be linked with lower perceived social isolation [58], and part of this benefit may come through engagement with others.

Third sector staff and the LEAG emphasised the importance of creating a variety of accessible ‘safe spaces’ meeting different needs and preferences, including the non-neurotypical social and communication preferences of people with ASD [28, 59–61]. Online spaces such as Facebook were not always considered ‘safe’ by young people [51], and more moderated and specific online spaces may be required (e.g. [55]). Third sector sources also advocated addressing bullying to reduce youth loneliness [62–64].

### Mechanisms of action: intrapersonal

‘Changing thinking patterns and behaviour’, for example in relation to negative self-perceptions and withdrawal, may be a key mechanism in addressing chronic loneliness. A group intervention for high-schoolers with depression included a focus on replacing negative cognitions with positive ones, as well as on increasing participant involvement in pleasant activities [42]. Furthermore, the quantitative association found between loneliness and negative attitudes towards aloneness [52] suggests that reframing such thinking might be a potential intervention target. We did not find interventions focusing on changing social cognitions, such as interpreting ambiguous social stimuli as threatening, despite theoretical grounds for expecting such interventions to be promising [33].

Another potential psychological mechanism was ‘Building a positive attitude to oneself’, which was given preliminary support as a plausible mechanism by the qualitative literature and was emphasised by the LEAG. Associated qualitative themes included greater self-awareness [47], self-reliance [48], self-confidence [49], and self-efficacy [56].

Creating a sense of ‘purpose, ownership and control’ might counteract feelings of helplessness about chronic loneliness (LEAG). For example, the CBT-based online Entourage platform uses bespoke therapy comics to help users with social anxiety work towards their goals (e.g. attending a party) using a strength-based approach, alongside support from e-mentors (trained clinicians and peer mentors) who provide opportunities for social connectedness [38, 39].

### Mechanisms of action: interpersonal

‘Building social skills’ and ‘building social confidence’ are plausible interpersonal mechanisms for reducing loneliness. For instance, participants in an intervention for ASD university students reported:

“Well I figured out…how to change my social skills and little bits and pieces that I didn’t know were actually very negative.” ([54]: page 25)

“For the first time in my life, my friends from group and I went to [coffee shop]…I’ve had good opportunities from this group to practice good social skills and how to apply them elsewhere.” ([54]: page 25)

A similar increase in social confidence was echoed for an intervention for homeless youth:

“I’m a bit more outgoing and, like, I’ll go do more things now. I’m not so shy. I used to be really shy. (19-year-old)” ([56]: page 70)

### Mechanisms of action: social

Having meaningful companionship seems to be a key way to alleviate loneliness [48, 51, 56, 65]. For instance, although social media can be seen as a useful way to maintain contact with family and friends,

[the] sense of connectedness to the world through Facebook dissipates if people cannot establish meaningful communication, beyond greetings. ([51]: page 11)

The importance of ‘consistent social support’ from a relatable adult to build trust was highlighted by third sector staff [28] and in the published literature ([49]: page 182):

“… it was incredibly nice to have an adult I could call when I wanted…”

The Social Mechanisms construct ‘Recognising the shared understanding of peers’ was strongly supported for in-person and online group activities, and relevant to medical students [47], hospitalised youth [27], young people with a parent suffering mental ill-health [49], youth not in education or employment [55], and university students with depression [36] or ASD [54].

“I think it just makes me feel better, just knowing there’s people out there just like me [with ASD]…I know I have people to talk to and people that I can ask for support”. ([54]: page 25)

In evaluating their intervention, [55] noted that their Moderated Anonymous Online Groups (MAOGs) should be specific to both location and the young people’s situation, for example having shared experiences of being bullied. Communicating about shared experiences might overcome the barrier of ‘not talking about loneliness’ identified by third sector staff [28, 29] and reported for homeless youth [65] and students [48]. Finding commonality and belonging with others is likely to help ‘create meaningful relationships’ and ‘build a sense of community’, as well as potentially addressing psychological barriers such as mistrust.

Activities that ‘build social identity’, such as music therapy ([27]: page 94) or activities that facilitate shared family identity [66], could plausibly reduce loneliness through increasing feelings of belonging. For instance, a peer-support group for ASD university students facilitated identity-building:

“Trying to find who I am. Trying to figure out my identity. Even with the ASD, the spectrum disorder, knowing that I can pretty much do anything that anyone else can. I just have a back-up system [the support group].” ([54]: page 25)

A number of third sector and policy sources advocated training of parents, educators, service providers and community members to improve understanding of loneliness and specific needs, for example associated with disability, mental ill-health or particular social and communication needs, as well as anti-bullying campaigns [62–64, 67–70]. The LEAG proposed addressing familial, community and societal stigma related to loneliness and mental ill-health as an important backdrop to individual-level strategies.

### Barriers

Individual hurdles probably mediate whether the strategies outlined above are effective. For example, a mentoring scheme would be inappropriate for someone who is housebound with severe anxiety or depression, but might suit someone with milder symptoms. Individual hurdles to addressing chronic loneliness may include psychological barriers such as not wanting to be a burden and feeling that others do not share the same experiences [48, 49], as well as situational factors such as caring responsibilities or work patterns (LEAG). The LEAG also raised being a refugee as being both a risk factor for loneliness (as also reported by [37]) and a potential barrier to addressing loneliness: for instance, due to language barriers creating challenges to accessing information and engaging with available support and activities, as well as a potential lack of access to employment and the social networks that work can provide, or the financial resources to engage with community activities that provide opportunities for social interaction. Qualitative data suggested that receiving professional therapy might help overcome the barrier of not wanting to be a burden:

“Having somebody external that didn’t know me personally so that I didn’t feel guilty about telling them about what was going on would have really helped me to be able to talk about what I was feeling…” ([48]: page 24)

Broader practical hurdles include the inaccessibility of services and community assets related to transport, finances, disability, neurodiversity, waiting times, and the digital divide, as well as whether an individual’s interests are catered for locally [59–62,68]. It is likely that digital exclusion has presented a substantial barrier during the current COVID-19 pandemic. Social hurdles include stigma of both loneliness and mental ill-health, which relates to the tendency to not discuss loneliness [28, 65], lack of understanding from service providers [59–61], and unsupportive home environments (LEAG, [29]).

### Which aspects of interventions may be most effective, and in which combinations?

Through a CIS approach incorporating RRR principles we developed a conceptual framework that can be used to generate testable hypotheses about which strategy(s) might work best for whom and why. The conceptual framework proposes possible pathways through which particular “Context” factors might influence which “Content” is most effective for which group of young people under which circumstances. For instance, it is plausible that if loneliness primarily arises from psychological barriers including anxiety or depression, then therapy may be most effective in reducing loneliness, acting through intrapersonal mechanisms such as changing thinking and behaviour that help build more positive attitudes to self and others and which feed back into reduced anxiety and depression (the ‘Intrapersonal’ pathway). In contrast, if an individual would like support building communication skills or confidence, for example due to ASD, interventions focusing on these needs may be more effective (‘Interpersonal’ pathway). Lacking close relationships might be best addressed through enhancing social support via peer mentors or support groups, whereas a lack of belonging might be alleviated through shared activities such as music-making or sports, all of which can help individuals recognise commonality and build connections with others (‘Social’ pathway).

To complement the CIS-derived framework, in this section we outline the current state of the evidence for the effectiveness of interventions in these ‘Intrapersonal’, ‘Interpersonal’ and ‘Social’ pathways. Convincing assessment of the effectiveness requires fully-powered RCTs. Only nine of the 27 included studies (33%) were randomised controlled trials [12, 13, 16, 42, 44, 45, 52, 53, 55]. Of these, only two report power calculations [16, 44]. First, Rohde et al. ([44]: *n* = 109] report an effect size calculation, with this pilot study being powered to detect medium to large effect sizes. However, no significant difference between the CBT Coping Course treatment and control groups of incarcerated young men was found for loneliness, and the significantly greater improvements in externalising scores, self-esteem and reduced suicide-proneness in the treatment group compared to controls showed only small effects sizes (Table 2). Second, Larsen et al. [16] indicate in their study protocol [71] that a sample of 975 students and 49 classes was needed to detect a small effect size of 0.25. The retained sample size of 1937 high school students in their study suggests that this trial is potentially adequately powered, but they do not report how many classes participated and in their discussion of study limitations the authors report lack of statistical power due to the low number of participating schools (*n* = 17 schools), since the analyses were adjusted for the clustered structure of the data. This study found no effect of the school-based intervention on students’ mental health problems or loneliness, and severity actually increased in all conditions [16] (Table 2). However, girls in the multi-tier group, who received professional support with mental health in addition to having peer mentors and class-based activities that aimed to enhance the psychosocial environment of the school, had a significantly smaller increase in mental health problems compared to girls in the control group [16]. Both these studies were quality rated as ‘fair’. In summary, the two RCTs that appear to have been sufficiently powered found no significant effect of either intervention on loneliness, thus yielding no evidence for the effectiveness of ‘Intrapersonal’ (CBT Coping Training) or ‘Social’ (improved social support in schools) strategies.

Of the RCTs that did not explicitly report sufficient power, four primarily involved Intrapersonal Strategies (Tables 1 and 2). First, undergraduate psychology students with moderate depression receiving a “reframing” intervention were found to experience greater reductions in depressive symptoms than those in “self-control” intervention or control conditions, but loneliness was found to decrease over time irrespective of condition [12]. The sample size for this study was *n* = 57 and it was quality rated as ‘fair’; no effect sizes were reported. Second, it was found that quitting social media sites did not change social or emotional loneliness compared to controls continuing use as usual [52]. However, this study was quality rated as ‘poor’ (*n* = 77). Moreover, this finding contrasts with an earlier RCT [72], which found that in a sample of undergraduates (*n* = 143) reduced use, rather than complete cessation, of Facebook, Instagram and Snapchat led to a greater reduction in loneliness and depression than in a ‘behaviour as normal’ control group (please note that this paper was not included in our initial analysis because our quantitative searches aimed to update Loades et al. [14] and did not include papers before 2020—we thank an anonymous reviewer for bringing this paper to our attention). Third, loneliness significantly decreased in adolescents at risk of alcohol and marijuana use presenting at primary care clinics receiving peer network counselling compared to active controls in a study quality rated as ‘good’ and with a sample size over 100 (*n* = 117), albeit with a minimal effect size [45]. Fourth, CBT yielded greater reductions in loneliness and depressive symptoms in a group of at-risk adolescents with elevated depression symptoms compared to controls with no effect size reported and a small effect size, respectively, in a ‘good’ quality study with a relatively large sample size (*n* = 341) [42]. While the findings were mixed regarding Intrapersonal Strategies, it is worth noting that both studies with sample sizes over 100, which were both quality rated as ‘good’, found significant decreases in loneliness after peer network counselling or CBT compared to controls, although effect sizes were small or not reported [42, 45].

Two further RCTs examined ‘social skills’ training interventions (Table 2). The first was quality rated as ‘fair’ but the authors explicitly identified lack of statistical power as a limitation of their study, and present their findings as preliminary findings from a pilot study: in a group of adolescents with ASD, they found large effect sizes for reductions in loneliness and improvements in ‘social skills’ after social skills training with or without peer supporters compared to waiting list controls ([53]: *n* = 34]. In the second study, which was quality rated as ‘good’, a similar sample size was used (*n* = 35), suggesting that this can also be considered a pilot study: moderate to strong effect sizes were found for greater reductions in social anxiety in participants receiving social skills training compared to waitlist controls, but no difference in loneliness was found between conditions over time [13]. Pilot findings are therefore mixed regarding Interpersonal Strategies, with some suggestion that ‘social skills’ training maybe particularly useful for young people with ASD in addressing their loneliness.

Regarding Social Strategies, in a quasi-experimental study in which young adults not in employment or education were randomly allocated to either join a moderated anonymous online group or not, no significant changes in quality of life or loneliness were detected ([55]: *n* = 147; quality rated as “fair”) (Table 1). Alongside the apparently well-powered school-based RCT described above [16] (Table 2), which did not find any effect on loneliness or mental health of class-based activities, peer mentors or a professional mental health support team, this yields no evidence so far of the effectiveness of Social Strategies for addressing loneliness in young people.

## Discussion

Based on current evidence, the new framework provides exploratory insights into what might help address loneliness in particular contexts and why. The framework should be seen as a provisional library of potential strategies that researchers, in collaboration with young people, clinicians and policy-makers, can use to co-design, develop and test effective strategies for addressing loneliness as an active ingredient in preventing and alleviating anxiety and depression in young people. Interventions that flexibly combine Intrapersonal, Interpersonal and Social approaches may be particularly effective: for example, Entourage combines an individualised online therapeutic platform with e-mentor support [38, 39]. Further development and evaluation of approaches that provide both social support and psychological therapy (e.g. [39, 40]) is needed, as Intrapersonal and Social strategies may reinforce one another [40]. Discussion with the LEAG indicated that a certain level of psychological health and confidence was required before engagement with social opportunities became viable, suggesting that Intrapersonal strategies may be a key gateway into other approaches.

The framework builds on previous taxonomies of interventions for loneliness [21, 22], which only incorporate a classification of the ‘Content’ of strategies to address loneliness. The purpose of this current review was to also conceptualise both the contextual factors that may determine what individual-level strategies might work for whom, and the potential mechanisms of action that might explain why particular strategies work. Future work should seek to incorporate socio-political-level strategies as well, but this was beyond the scope of this current review. Our new conceptualisation of the Content of strategies to address loneliness maps straightforwardly on to Mann et al’s [22] categorisation of loneliness interventions for people with mental ill-health, indicating that this typology remains relevant in this specific age group of 14–24-year olds. In the new framework ‘Intrapersonal Strategies’ includes Mann et al.’s ‘changing cognitions’ but also includes psychoeducation, which Mann et al categorise along with ‘social skills training’. The broader label of ‘Intrapersonal Strategies’ used here also incorporates informal self-help strategies. ‘Interpersonal Strategies’ is used to describe Mann et al.’s ‘social skills training’ category. Mann et al. distinguish between ‘supported socialisation or having a socially-focused supporter’ and ‘wider community approaches’, which we have combined into ‘Social Strategies’. However, within our framework we continue to acknowledge this distinction through two sub-constructs that draw on Masi et al.’s classification [21]: strategies that ‘Enhance social support’ (e.g. involving peers, family, or relatable adults) may best serve deficits in close relationships, whereas ‘Increasing opportunities for social contact’ may best answer a ‘Sense of difference’ or lack of connection to the wider community. However, we combined these two approaches because we hypothesis that they likely act through common ‘Social Mechanisms’. These potential mechanisms can themselves be targeted in future intervention development.

We reviewed outcome findings for RCTs in order to assess effectiveness of these different types of strategies. The lack of reported power calculations for most studies limits the strength of the conclusions that can be drawn. Although findings were mixed, the most convincing evidence was found in support of Intrapersonal Strategies: two studies with sample sizes over 100, which were both quality rated as ‘good’ but did not report power calculations, found significant decreases in loneliness after peer network counselling (for adolescents at risk of alcohol and marijuana use presenting at primary care clinics) or CBT (in adolescents with elevated depression symptoms) compared to controls, although effect sizes were small or not reported [42, 45]; CBT was also found to decrease depressive symptoms [42]. However, a third RCT that seemed to be fully powered failed to find an effect of CBT Coping Training on loneliness in a sample of incarcerated young men despite finding improvements in externalising scores, suicide-proneness and self-esteem [44]. Pilot findings were also mixed regarding Interpersonal Strategies, with some suggestion that training on developing social skills maybe useful for young people with ASD in addressing their loneliness [53], but perhaps not those with social anxiety [13]. However, there was some concern in the LEAG that such interventions for ASD may promote only ‘neurotypical’ social skills and that societal attitudes and expectations also need to be addressed to help reduce loneliness in individuals with ASD. No evidence was found in support of the effectiveness of Social Strategies for addressing loneliness in young people [16, 55].

An important finding from this review is that creating opportunities for young people to engage with others with similar experiences is a key Social Mechanism for addressing loneliness, perhaps alongside more targeted social skills training (e.g. [54]: for university students with ASD, likely involving Interpersonal Mechanisms) or therapy to overcome psychological barriers such as self-stigma (e.g. [43]: for gay and bisexual young men, which may be transferable to other demographics, and likely involves Intrapersonal Mechanisms). Social skills and confidence may also develop inadvertently in group-based interventions, and social confidence may come not only from greater assurance in the individual’s own ability to socialise, but also in greater trust that others will respond positively. The most prominent social hurdle raised by the LEAG was stigma attached to both loneliness and mental ill-health (as well as stigma related to other experiences, such as having ASD or low socioeconomic status), which may hint at why ‘Recognising the shared understanding of peers’ seemed so powerful as a potential mechanism of action.

Despite the evidence for associations between loneliness and youth anxiety and depression [14], few studies directly tested whether reductions in loneliness also reduced anxiety or depression, or the mechanisms by which this might occur. Given the clear role of identity and sharing experiences in reducing loneliness, interventions such as Groups4Health [73, 74], which aim to build stronger social identities, might be particularly promising. No interventions for loneliness were found addressing societal stigma or incorporating the built environment, and these were flagged as important areas to address (LEAG, expert panel, [64, 67–70]). Given the importance of familial social support for adolescents [75], interventions to improve such relationships might also be helpful. Equally, cognitive biases such as hypersensitivity to social threat are known to be associated with loneliness [33], yet no psychological interventions were found addressing these specifically in relation to loneliness (i.e. with loneliness as a measured outcome) for this age group. There are likely to be a number of promising interventions that were not included in this review because they did not aim to target loneliness specifically but could nonetheless yield reductions in loneliness for young people, for instance through targeting a related social construct. One such promising intervention [76] aimed to modify social appraisals by targeting university students’ sense of belonging (a concept related to loneliness and part of the ‘Social’ pathways in the conceptual framework presented here). This study found that African American university students who were randomly assigned to an intervention in which they reframed feelings of not belonging as shared and transitory, being a natural part of starting at college rather than due to their minority status, were found to have improved health and wellbeing compared to controls [76]. Such findings suggest that changing social cognitions in this age group may also help in reducing at least social loneliness, which is linked to not feeling part of a wider community. Only one of the included studies specifically targeted young people who were lonely [48], whereas others recruited those who might be at risk of loneliness, and none of the studies distinguished chronic from transitory loneliness so our framework pertains to loneliness in general [17]. Broader community-level or societal approaches that aim to improve education attainment, raise household income and build neighbourhood assets may also have downstream effects on reducing loneliness and improving mental health, since these socioeconomic factors are associated with increased loneliness [37]. Future work could expand the conceptual framework presented here to incorporate such approaches, which were outside of the current remit, which was to identify potential approaches to incorporate in clinical and social interventions at the individual level.

A strength of this review was the novel methodology: we critically synthesised diverse strands of evidence collated through a robust and iterative search and extraction strategy involving independent raters, and incorporated rapid realist review principles to ensure policy and practice relevance. We included coping strategies as well as formal interventions to gain a wider perspective on what might help young people overcome loneliness. Another distinctive strength is that we not only consulted with young people with relevant lived experience but also a cross-disciplinary panel of academic experts that included perspectives from neuroscience, the built environment, arts and health, social work and digital technology, and which complements the strong clinical psychology and psychiatry expertise in our author team. However, some disciplines pertinent to loneliness in young people were not represented, such as sociology, social psychology and experts on complex interventions, which may have limited the scope of the evidence and biased the framework to some extent.

A limitation was that despite the iterative nature of our search strategy and consultation of experts from diverse disciplines, we may have missed relevant studies. In particular, we relied on three recent reviews [11, 14, 17], two of which were both published within 6 months of our searches [14, 17], to provide quantitative studies, which we supplemented with updated searches based on the protocol of the most recent review, which specifically looked at loneliness and mental health in young people [14]. A downside of this is that any quantitative papers that were missed in these previous reviews will also be missing from this one. Nonetheless, we employed consultation with academic experts to try to minimise the likelihood of missing key papers. We did not rely on previous reviews for published qualitative studies or grey literature. We also focused specifically on interventions to reduce loneliness, and therefore do not include interventions targeting related social constructions, which may also yield reduced loneliness. Broadening the proposed framework to include related social constructs, such as belonging, is an area for future research. We consulted 18–24-year olds and although LEAG members drew on their adolescent experiences, young adults may not be aware of current barriers and opportunities facing younger age groups in a rapidly changing social environment. CIS includes studies on the basis of relevance rather than quality, meaning the synthesised evidence was limited by study quality (Supplementary Table S2), particularly since we included grey literature evidence that was not peer-reviewed. However, constructs supported by academic studies were associated with at least one study rated as ‘fair’ or ‘good’ or with COREQ scores over 70% (Fig. 2). Furthermore, we focused our discussion of outcomes on RCTs as only this study design can contribute meaningfully to understanding the effectiveness of interventions. The limited number of good quality RCTs indicate that more fully-powered RCTs are required in relation to all the constructs. Nonetheless, by triangulating diverse academic evidence and a wealth of lived and professional experience, we developed a single coherent framework in order to facilitate researchers, practitioners and policy-makers in thinking about what might help or not help young people to address loneliness in different contexts, as an active ingredient in preventing or alleviating anxiety and depression.

### Commentary written by young people with lived experience

The LEAG agreed that improving loneliness can be an active ingredient in preventing and reducing youth depression and anxiety. Despite individual differences between whether loneliness or mental ill-health arose first, there was general agreement that loneliness and depression/anxiety are interlinked and can feed into each other.

The developed framework aligns with the experiences of the group and the co-designed, individualised construct in particular resonated with members, who emphasised the importance of personalised strategies. The LEAG highlighted that individuals should have more agency when engaging in mental health interventions, have their voices heard and challenge ideas provided from the services. The LEAG also expressed frustrations surrounding a lack of communication between services, highlighting the importance of transitions and treating the individual rather than a set of symptoms.

The group agreed that activities which build self-esteem, social skills and confidence are essential in reducing loneliness, but felt that practical and social barriers affect this: for example, lack of socioeconomic accessibility and stigma. The group identified these barriers as often occurring together, creating further obstacles in alleviating their loneliness. For those with chronic loneliness or depression, the experience may become the individual’s identity. As a result, treatments focusing on developing an alternative identity may be a promising avenue for reducing chronic loneliness.

Along with those presented in the study, having a meaningful job was added as providing purpose, helping reduce loneliness and improving mental health. Denial about illness and a lack of mental health interventions were also suggested as further intrapersonal barriers.

## Supplementary information

Supplementary Materials - methods & measures
