# Supplementary material for: Loneliness as an active ingredient in preventing or alleviating youth anxiety and depression: a critical interpretative synthesis incorporating principles from rapid realist reviews
Source: Transl Psychiatry. 2021 Dec 10;11:628. doi: 10.1038/s41398-021-01740-w (PMC8661314; doi:10.1038/s41398-021-01740-w)
Supplement: Supplementary file 1 — Supplementary Materials - methods & measures [file 41398_2021_1740_MOESM1_ESM.docx]

**Supplementary Materials**

**Loneliness as an active ingredient in preventing or alleviating youth anxiety and depression: a critical interpretative synthesis incorporating principles from rapid realist reviews**

**Supplementary methods**

The combined Critical Interpretive Synthesis-Rapid Realist Review (CIS-RRR) approach was well-suited to the research questions: it allowed the emerging theoretical framework to be informed by a reflexive approach to integrating findings from diverse literature alongside perspectives from academic and lived experience experts. This approach provided new insights and iterative reconceptualisation of the research questions to establish a critically informed theoretical framework grounded in the available evidence, using a practical, outcomes-focused knowledge synthesis method, over a short time period.

***Literature searches***

Searches (Table S1) were conducted from June 19^th^ to July 1^st^, 2020 using the following databases: MEDLINE, PsycINFO, Embase, Web of Science, ProQuest, Scopus, CINHAL, and the Cochrane Collaboration Library using MeSH terms wherever possible. EMCare was added to the qualitative search as its primary focus is on nursing and allied health literature. Theses were searched for using five databases: the British Libraries e-theses online service, Dart Europe, ProQuest, NDTL Global and OATD. Retrieval of grey literature followed recommendations from (1), with searches conducted via greylit, opengrey and Mednar, and websites were searched for relevant content. A random sample of authors from reference lists in selected papers was searched. A total of 1350 references were reviewed and as no relevant literature was found, further reference list searching was discontinued. All authors of included papers were forward searched to identify additional relevant publications.

**Table S1:** Search terms for published and grey academic literature

| *Search terms*  1. Paediatric   \| exp Adolescent/ or exp Child/ or exp Child, Preschool/ or exp Infant/ or exp Minors/ or exp Pediatrics/ \| \| --- \| \| (adolesc* or preadolesc* or pre-adolesc* or boy* or girl* or child* or infan* or preschool* or pre-school* or juvenil* or minor* or pe?diatri* or pubescen* or pre-pubescen* or prepubescen* or puberty or teen* or young* or youth* or school* or high-school* or highschool* or schoolchild* or school child*).tw,kf. \|   2. Social isolation/loneliness   \| exp social isolation/ \| \| --- \| \| exp Loneliness/lonel* \|   3. Mental health   \| mental health [added to the Loades et al terms] \| \| --- \| \| wellbeing/ or well-being [added to the Loades et al terms] \| \| anxiet*/ or anxious*/ or "anxiety disorder*".tw,kf. \| \| depress*/ or "internal* disord*"/ or "low mood".tw,kf. \| \| depressive disorder/ \| \| exp depression/ \| \| depress*.tw,kf. \| \| exp adjustment disorders/ \| \| adjustment disorder*.tw,kf. \| \| low mood.tw,kf. \| \| obsessive-compulsive disorder.tw,kf. \| \| stress disorders, traumatic/ \| \| stress disorders, post-traumatic/ \| \| trauma*.tw,kf. \| \| (((post-trauma* or posttrauma*) adj (stress)) or PTSD).tw,kf \| \| social anxiety, social anxiety disorder, social phobia [added to the Loades et al terms] \|   4. Qualitative literature   \| (semi-structured or semistructured or unstructured or informal or in-depth or indepth or  face-to-face or structured or guide) or (interview* or discussion* or questionnaire*) or (focus group* or qualitative or ethnograph* or fieldwork or field work or key informant) or interviews as topic/ or focus groups/ or narration/ or qualitative research/ \| \| --- \| |
| --- | --- | --- | --- | --- | --- | --- | --- | --- | --- | --- | --- | --- | --- | --- | --- | --- | --- | --- | --- | --- | --- |

Third sector information was searched for (SP) using the terms ‘loneliness’, ‘social isolation’, and ‘children and young people’ via the google web search engine.

***Inclusion and Exclusion Criteria***

For inclusion, quantitative and qualitative studies required: loneliness as a primary or secondary outcome in the context of anxiety, depression, or ‘mental health’ (including wellbeing), publication in English in a peer-reviewed journal, have a mean age within the 14-24 years range, and include an intervention or coping strategy addressing loneliness. Grey literature was included along similar lines, without the publication criterion. Articles were excluded that did not: investigate loneliness, depression, anxiety or ‘mental health’ (broadly defined to include wellbeing), fit the age range, or include an intervention or strategy addressing loneliness.

***Screening process***

All records were uploaded to Rayyan (<https://rayyan.qcri.org/>) and duplications removed, followed by title and abstract screening (DS, EH, PMH, SO). Twenty-two potential studies were found: 20 from the primary search, one from forward searching of paper authors, and one from the targeted search focusing on eating disorders and stigma. Articles were included according to the specified criteria. This stage was conducted by four reviewers (DC, EH, PMH, SO). Studies were only removed at this stage if they had been reviewed by at least two reviewers (EH, PMH). Disagreements were resolved through discussion. Four of the 22 articles where disagreement could not be resolved were discussed with lead reviewers (EP, RS) and rejected. Of the excluded papers from the primary search, 20% were checked to ensure adherence to the eligibility criteria (DC, EH, PMH, SO). The 18 included papers were checked by at least two reviewers (EH, PMH).

***Quality assessment***

The Consolidated Criteria for Reporting Qualitative Research (COREQ: 2) rating scale was used to assess the quality of included qualitative studies by two reviewers (DS, SO): raw scores are given as a proportion of the total number of COREQ items relevant to the study (the highest possible score is 32). We did not convert this into a poor/fair/good classification as the COREQ does not provide instructions of how to translate raw scores into such a scoring categorisation.

Consistent with (3), a shortened quality assessment using criteria adapted from the National Institutes for Health (NIH) was used to assess the quality of included quantitative studies by one reviewer (EH). A fourth reviewer (PMH) checked all ratings and final ratings of ‘poor’, ‘fair’ and ‘good’ were agreed jointly by two reviewers (EH, PMH) (Table S2).

**Table S2:** Quality ratings for the 27 papers included in the CIS

| **Publication** | **Quality Measure** | **Quality Score** |
| --- | --- | --- |
| **Qualitative** | | |
| Dagan and Yager (2019) (4) | COREQ | 8/9 |
| Park (2012) (5) | COREQ | 26/30 |
| Rew (2000) (6) | COREQ | 24/26 |
| Rice et al. (2020a) (7) | COREQ | 12/21 |
| Ştefăniţiă, et al. (2018) (8) | COREQ | 17/31 |
| Trondsen (2012) (9) | COREQ | 26/29 |
| Vasileiou, et al. (2019) (10) | COREQ | 23/31 |
| **Mixed Method** | | |
| Agadullina et al. (2020) (11) | COREQ | 19/32 |
|  | NIH | Poor |
| Gold et al. (2019) (12) | COREQ | 20/23 |
|  | NIH | Poor |
| Hillier et al. (2018) (13) | COREQ | 25/32 |
|  | NIH | Fair |
| Horgan et al. (2013) (14) | COREQ | 20/32 |
|  | NIH | Poor |
| Kivijärvi et al. (2019) (15) | COREQ | 20/32 |
|  | NIH | Fair |
| Lim et al. (2019) (16) | COREQ | 26/32 |
|  | NIH* | Poor |
| Stewart et al. (2009) (17) | COREQ | 21/30 |
|  | NIH | Fair |
| **Quantitative** | | |
| Bernhold (2019) (18) | NIH | Poor |
| Conoley et al (1985) (19) | NIH | Fair |
| dos Santos et al. (2020) (20) | NIH | Fair |
| Kneer et al. (2019) (21) | NIH* | Fair |
| Larsen et al. (2019) (22) | NIH* | Fair |
| Mahdavi et al. (2020) (23) | NIH | Poor |
| Masia-Warner et al. (2005) (24) | NIH* | Good |
| Mason et al. (2016) (25) | NIH* | Good |
| Matthews et al. (2018) (26) | NIH* | Fair |
| Rice et al. (2020b) (27) | NIH | Fair |
| Rohde et al. (2004) (28) | NIH* | Fair |
| Smith et al. (2017) (29) | NIH* | Fair |
| Stice et al. (2010) (30) | NIH* | Good |
| COREQ (2): 32-item checklist for qualitative research. COREQ does not provide a scoring system of poor/fair/good so raw scores are given. Maximum score = 32. Maximum scores adjusted when items were not applicable. NIH short form used in (3). NIH* rated for quality in (31). | | |

**Measures of loneliness**

Of the 27 studies included in the synthesis, 13 used a form of the UCLA Loneliness Scale (Table S3). Six studies used interviews/forums/focus groups (5,6,8–10,27), one used no measure (4), the remaining seven each used different measures; three of which included a question recommended by the ONS (Table S4). One study (15) also included this question as well as a version of the UCLA Loneliness Scale. Other measures used included Peer loneliness (21); Norway Loneliness Scale (22); Multidimensional inventory of loneliness experience (MILE) (11); dos Santos et al (20) included 1 item: “In the past 12 months, how often have you felt alone?” rated as: “Never,” “Rarely,” “Sometimes,” “Most of the time,” and “Always”.

Six studies used the revised UCLA 20-item version (12,16,17,19,26,27): Table S3 (32). Three studies (13,23,29) used Version 3 of the 20-item UCLA loneliness scale with similar items phrased as questions e.g. ‘How often do you feel that you are "in tune" with the people around you?’ (33). Two studies used the 8-item version (28,30) and one study used the 3-item version (18), and one study used a 12-item version (15): Table S3. The 20-item UCLA loneliness scale (32) has good internal and test-retest reliability, as well as validity in terms of correlating with other loneliness measures and related factors such as wellbeing (33). This scale was developed in adults rather than adolescents, and measures loneliness in terms of frequency rather than intensity or duration.

**Table S3**: Items in the Revised UCLA Loneliness Scale (32) as well as 3-item, 8-item and 12-item versions.

| 1. I feel in tune with the people around me. |
| --- |
| 2. I lack companionship.* ** |
| 3. There is no one I can turn to.* |
| 4. I do not feel alone. |
| 5. I feel part of a group of friends. *** |
| 6. I have a lot in common with the people around me. *** |
| 7. I am no longer close to anyone. *** |
| 8. My interests and ideas are not shared by those around me. |
| 9. I am an outgoing person.* |
| 10. There are people I feel close to.* ** *** |
| 11. I feel left out. *** |
| 12. My social relationships are superficial. *** |
| 13. No one really knows me well. *** |
| 14. I feel isolated from others.* ** *** |
| 15. I can find companionship when I want it.* *** |
| 16. There are people who really understand me. *** |
| 17. I am unhappy being so withdrawn.* *** |
| 18. People are around me but not with me.* |
| 19. There are people I can talk to. *** |
| 20. There are people I can turn to. |
| Items rated as: Never, Rarely, Sometimes, Often. Scoring: Items 1, 5, 6, 9, 10, 15, 16, 19, 20 are all reverse scored. *8-item version **3-item version ***12-items used by (15) |

The Office of National Statistics (ONS) recommends use of both the 3-item UCLA loneliness scale (18) and a single item direct measure of loneliness (Table S4 for adults, Table S5 for children) (34). In total 13 studies included the three UCLA Loneliness Scale items, mostly as part of longer scales (see text above and Table S3). Two studies (14,15) included the direct measure of loneliness suggested by the ONS. The Loneliness Scale used by (24) included “I’m lonely” rated on a 5-point Likert scale from “not true at all” to “always true”. One study (25) included “I am lonely” rated as true/false.

**Table S4: ONS recommended measures of loneliness for adults** (34)

| **Measures** | **Items** | **Response categories** |
| --- | --- | --- |
| The three-item UCLA Loneliness scale | 1.     How often do you feel that you lack companionship? | Hardly ever or never, Some of the time, Often |
|  | 2.     How often do you feel left out? | Hardly ever or never, Some of the time, Often |
|  | 3.     How often do you feel isolated from others? | Hardly ever or never, Some of the time, Often |
| The direct measure of loneliness | How often do you feel lonely? | Often/always, Some of the time, Occasionally, Hardly ever, Never |

**Table S5: ONS recommended measures of loneliness for children** (34)

| **Measures** | **Items** | **Response categories** |
| --- | --- | --- |
| The three -item UCLA Loneliness scale for children | 1.      How often do you feel that you have no one to talk to? | Hardly ever or never, Some of the time, Often |
|  | 2.      How often do you feel left out? | Hardly ever or never, Some of the time, Often |
|  | 3.      How often do you feel alone? | Hardly ever or never, Some of the time, Often |
| The direct measure of loneliness | How often do you feel lonely? | Often/always, Some of the time, Occasionally, Hardly ever, Never |

**Measures of anxiety and depression**

Depression symptoms were measured using a number of different validated scales. The most commonly used measure was the Centre for Epidemiological Studies – Depression scale (CES-D) (35), which was used by four of the included studies (14,16,17,29). One further study (18) used a shortened 6-item version of the CES-D scale but reference the original 20-item version (35). The next most common measure of depression symptoms was the Beck Depression Inventory (36), which was used by two included studies (19,30). All other measures were used by single studies: the Patient Health Questionnaire-9 (37) and the Male Depression Risk Scale (38) were used by (27); the Children’s Depression Inventory (39) was used by (24); and two-items from the Behavior Assessment System for Children (40) was used to measure depression symptoms by (25). One study (28) used the Youth Self-Report scale (41) to measure internalising and externalizing behaviours, as well as the Current Suicidal ideation and Lifetime Suicidal Attempts scale (42).

Social anxiety symptoms were also measured using a variety of validated scales. The most common scales for social anxiety were the Social Interaction Anxiety Scale (SIAS) and the Liebowitz Social Anxiety Scale. Two studies (26,27) used the Social Interaction Anxiety Scale (SIAS) (43), and one further study (16) used a shortened form of this scale (S-SIAS), following findings that using the 17 of the 20 items that are more straightforwardly worded is a more valid indicator of social interaction anxiety than also using the reverse-scored items (44). The Liebowitz Social Anxiety Scale (45) was used by (27) and a version of this scale adapted for use with children and adolescents (46) was used by (24). One study (21) used a 9-item measure of social anxiety developed by (47) and another study (27) used the Brief Fear of Negative Evaluation from Others Scale (48). One study (24) used the Social Phobic Disorders Severity and Change Form (49), the Social Phobia and Anxiety Inventory for Children (50) and the Social Anxiety Scale for Adolescents in both self-report (51) and parent (51) versions, as well as the Anxiety Disorders Interview Schedule for DSM IV: Parent and Child Versions (52) to ensure participants met diagnostic criteria for social anxiety disorder. Another study reported the development of a digital intervention for young people with Social Anxiety disorder (SAD), which included consultation with young people with ‘lived experience of SAD’: no details are given on whether members of the participatory design forums were required to meet diagnostic criteria and if so how this was discerned (7).

One study measured anxiety more generally: (29) used the State-Trait Inventory for Cognitive and Somatic Anxiety (53). A second study (27) used a more general measure of anxiety, the Anxiety Sensitivity Index (54), but aimed to do so in order to measure social anxiety, which was also measured using more specific scales (see above).

Three studies used measures that relate to both anxiety and depression symptoms or associated issues. One study (13) used four of the seven sub-scales of the Counseling Center Assessment of Psychological Symptoms-34 Scale (55) to measure symptoms of depression, generalized anxiety, social anxiety, and academic distress. A second study (22) measured joint symptoms of anxiety and depression through the short form of the Symptom Check List (SCL-5) (56), which aims to measure global mental distress. A third included study (23) used the Emotion Regulation Difficulty Questionnaire (57), which aims to measure emotion dysregulation, which may underlie both anxiety and depression.

**References**

1. Aromataris EC, Ritano D. Constructing a search strategy and searching for evidence. A guide to the literature search for a systematic for a systematic review. Am J Nurs. 2014;14(5):49–56.

2. Tong A, Sainsbury P, Craig J. Consolidated Criteria for Reporting Qualitative Research (COREQ): A 32-Item Checklist for Interviews and Focus Groups. Int J Qual Heal Care. 2007;19:349–57.

3. Loades ME, Chatburn E, Higson-Sweeney N, Reynolds S, Shafran R, Brigden A, et al. Rapid Systematic Review: The Impact of Social Isolation and Loneliness on the Mental Health of Children and Adolescents in the Context of COVID-19. Journal of the American Academy of Child & Adolescent Psychiatry. 2020.

4. Dagan Y, Yager J. Addressing Loneliness in Complex PTSD. J Nerv Ment Dis. 2019;207(6):433–9.

5. Park MM. The experience of music therapy among adolescents at a children’s hospital in the San Francisco Bay area: A qualitative exploration. John F. Kennedy University; 2012.

6. Rew L. Coping With Loneliness Among Homeless Youth. J Child Adolesc Psychiatr Nurs. 2000;13(3):125–40.

7. Rice S, O’Bree B, Wilson M, McEnery C, Lim MH, Hamilton M, et al. Development of a graphic medicine-enabled social media-based intervention for youth social anxiety. Clin Psychol. 2020;(September 2019):1–13.

8. Ștefăniță O, Udrea G, Durach F, Corbu N. Facebook Use Among Romanian Graduate Students. Influences on Self-esteem and Feelings of Loneliness. J Media Res. 2018;11(1 (30)):5–19.

9. Trondsen M V. Living with a mentally Ill parent: Exploring adolescents’ experiences and perspectives. Qual Health Res. 2012;22(2):174–88.

10. Vasileiou K, Barnett J, Barreto M, Vines J, Atkinson M, Long K, et al. Coping with loneliness at University: A qualitative interview study with students in the UK. Ment Heal Prev. 2019;13:21–30.

11. Agadullina ER, Lovakov A, Kiselnikova N V. Does quitting social networks change feelings of loneliness among freshmen? An experimental study. J Appl Res High Educ. 2020;

12. Gold JA, Bentzley JP, Franciscus AM, Forte C, De Golia SG. An Intervention in Social Connection: Medical Student Reflection Groups. Acad Psychiatry. 2019;43(4):375–80.

13. Hillier A, Goldstein J, Murphy D, Trietsch R, Keeves J, Mendes E, et al. Supporting university students with autism spectrum disorder. Autism. 2018;22(1):20–8.

14. Horgan AM, McCarthy G, Sweeney JF. An evaluation of an online peer support forum for university students with depressive symptoms. Arch Psychiatr Nurs. 2013;27:84–9.

15. Kivijärvi A, Aaltonen S, Välimäki V. The feasibility of an online discussion group as a component of targeted youth work in Finland. Child Youth Serv Rev. 2019;105(March):104411.

16. Lim MH, Rodebaugh TL, Eres R, Long KM, Penn DL, Gleeson JFMM. A Pilot Digital Intervention Targeting Loneliness in Youth Mental Health . Front Psychiatry [Internet]. 2019;10(August):604. Available from: https://www.frontiersin.org/article/10.3389/fpsyt.2019.00604

17. Stewart M, Reutter L, Letourneau N, Makwarimba E. A Support Intervention to Promote Health and Coping Among HomelessYouths. CJNR 2009,Vol. 2009;41(2):54–77.

18. Bernhold QS. Grandparents’ Affectionate Communication toward Grandchildren and Grandchildren’s Mental Health Difficulties: The Moderating Role of Future Time Perspective. Health Commun. 2020;35(7):822–31.

19. Conoley CW, Garber RA. Effects of reframing and self-control directives on loneliness, depression, and controllability. J Couns Psychol. 1985;32(1):139–42.

20. dos Santos AE, Araujo RH de O, Nascimento VMS do, Couto J de O, Silva RJ dos S. Associations between specific physical activity domains and social isolation in 102,072 Brazilian adolescents: Data from the 2015 National School–Based Health Survey. J Health Psychol. 2020;

21. Kneer J, Van Eldik AK, Jansz J, Eischeid S, Usta M. With a little help from my friends: Peer coaching for refugee adolescents and the role of social media. Media Commun. 2019;7(2):264–74.

22. Larsen TB, Urke H, Tobro M, Årdal E, Waldahl RH, Djupedal I, et al. Promoting Mental Health and Preventing Loneliness in Upper Secondary School in Norway: Effects of a Randomized Controlled Trial. Scand J Educ Res. 2019;0(0):1–14.

23. Mahdavi A, Yaghoobi A, Rashid K, Kordnoghabi R. Comparison of the Effect of Compassion based Techniques and Cognitive Behavioral Schema Therapy Techniques in Reducing Loneliness and Emotion Regulation Difficulties in Runaway Adolescent Girls. Iran Evol Educ Psychol J. 2020;2(1):33–45.

24. Masia-Warner C, Klein RG, Dent HC, Fisher PH, Alvir J, Albano AM, et al. School-based intervention for adolescents with social anxiety disorder: Results of a controlled study. J Abnorm Child Psychol. 2005;33(6):707–22.

25. Mason MJ, Zaharakis NM, Sabo R. Reducing Social Stress in Urban Adolescents with Peer Network Counseling. J Child Fam Stud. 2016;25(12):3488–96.

26. Matthews NL, Orr BC, Warriner K, DeCarlo M, Sorensen M, Laflin J, et al. Exploring the Effectiveness of a Peer-Mediated Model of the PEERS Curriculum: A Pilot Randomized Control Trial. J Autism Dev Disord. 2018;48(7):2458–75.

27. Rice S, O’Bree B, Wilson M, McEnery C, Lim MH, Hamilton M, et al. Leveraging the social network for treatment of social anxiety: Pilot study of a youth-specific digital intervention with a focus on engagement of young men. Internet Interv. 2020;20(April):100323.

28. Rohde P, Jorgensen JS, Seeley JR, Mace DE. Pilot evaluation of the coping course: A cognitive-behavioral intervention to enhance coping skills in incarcerated youth. J Am Acad Child Adolesc Psychiatry. 2004;43(6):669–76.

29. Smith NG, Hart TA, Kidwai A, Vernon JRG, Blais M, Adam B. Results of a Pilot Study to Ameliorate Psychological and Behavioral Outcomes of Minority Stress Among Young Gay and Bisexual Men. Behav Ther. 2017;48(5):664–77.

30. Stice E, Rohde P, Seeley JR, Gau JM. Testing Mediators of Intervention Effects in Randomized Controlled Trials: An Evaluation of Three Depression Prevention Programs. J Consult Clin Psychol 2010. 2010;78(2):273–80.

31. Eccles AM, Qualter P. Review: Alleviating loneliness in young people – a meta-analysis of interventions. Child Adolesc Ment Health. 2020;

32. Russell, D., Peplau, L. A., & Cutrona CE. The revised UCLA Loneliness Scale: Concurrent and discriminant validity evidence. J Pers Soc Psychol. 1980;39:472–80.

33. Russell D. UCLA Loneliness Scale (Version 3): Reliability, validity, and factor structure. J Pers Assess. 1996;66(1):20–40.

34. Office of National Statistics. Measuring loneliness: guidance for use of the national indicators on surveys. 2018.

35. Radloff LS. The CES-D Scale: A Self-Report Depression Scale for Research in the General Population. Appl Psychol Meas. 1977;1(3):385–401.

36. Beck AT, Steer RA, Carbin MG. Psychometric properties of the Beck Depression Inventory: Twenty-five years of evaluation. Clin Psychol Rev [Internet]. 1988;8(1):77–100. Available from: https://www.sciencedirect.com/science/article/pii/0272735888900505

37. Kroenke K, Spitzer RL, Williams JB. The PHQ-9: validity of a brief depression severity measure. J Gen Intern Med. 2001/09/15. 2001;16(9):606–13.

38. Rice SM, Fallon BJ, Aucote HM, Möller-Leimkühler AM. Development and preliminary validation of the male depression risk scale: Furthering the assessment of depression in men. J Affect Disord [Internet]. 2013;151(3):950–8. Available from: http://dx.doi.org/10.1016/j.jad.2013.08.013

39. Kovacs M, Beck AT. An empirical–clinical approach toward a definition of childhood depression. In: Schulterbrandt JG, Raskin A, editors. Depression in childhood: Diagnosis, treatment, and conceptual models. New York: Raven; 1977. p. 1–25.

40. Reynolds CR, Kamphaus RW. BASC-2: Behavior assessment system for children. 2nd ed. Bloomington, MN: Bloomington, MN: Pearson; 2004.

41. TM A. Manual for the Youth Self-Report and 1991 Profile. Burlington: University of Vermont Department of Psychiatry; 1991.

42. Lewinsohn PM, Rohde P, Seeley JR. Adolescent suicidal ideation and attempts: Prevalence, risk factors, and clinical implications. linical Psychol Sci Pract. 1996;3(1):25–46.

43. Mattick RP, Clarke JC. Development and validation of measures of social phobia scrutiny fear and social interaction anxiety. Behav Res Ther. 1998;36(4):455–70.

44. Rodebaugh TL, Heimberg RG, Brown PJ, Fernandez KC, Blanco C, Schneier FR, et al. More Reasons to be Straightforward: Findings and Norms for Two Scales Relevant to Social Anxiety. Anxiety Disord [Internet]. 2011;25(5):623–30. Available from: https://www.ncbi.nlm.nih.gov/pmc/articles/PMC3624763/pdf/nihms412728.pdf

45. Liebowitz MR. Social phobia. Klein D, editor. Anxiety Mod Trends Pharmacopsychiatry, Vol 22. 1987;22:141–73.

46. Masia-Warner C, Storch EA, Pincus DB, Klein RG, Heimberg RG, Liebowitz MR. The Liebowitz Social Anxiety Scale for Children and Adolescents: An initial psychometric investigation. J Am Acad Child Adolesc Psychiatry [Internet]. 2003;42(9):1076–84. Available from: http://dx.doi.org/10.1097/01.CHI.0000070249.24125.89

47. Crick NR, Ladd GW. Children’s perceptions of their peer experiences: Attributions, loneliness, social anxiety, and social avoidance. Dev Psychol. 1993;29(2):244–54.

48. Leary MR. A Brief Version of the Fear of Negative Evaluation Scale. Personal Soc Psychol Bull [Internet]. 1983 Sep;9(3):371–5. Available from: https://doi.org/10.1177/0146167283093007

49. Liebowitz MR, Schneier F, Campeas R, Hollander E, Hatterer J, Fyer A, et al. Phenelzine vs Atenolol in Social Phobia: A Placebo-Controlled Comparison. Arch Gen Psychiatry [Internet]. 1992 Apr 1;49(4):290–300. Available from: https://doi.org/10.1001/archpsyc.49.4.290

50. Beidel DC, Turner SM, Morris TL. A new inventory to assess childhood social anxiety and phobia: The Social Phobia and Anxiety Inventory for Children. Psychol Assess. 1995;7(1):73–9.

51. LaGreca AM. Social anxiety scales for children and adolescents: Manual and instructions for the SASC, SASC-R, SAS-A, and parent versions of the scales. 1998.

52. Silverman WK, Albano AM. Anxiety Disorders Interview Schedule for DSM-IV-Child and Parent Versions. San Antonio, TX: Graywind, A Division of the Psychological Corporation.; 1996.

53. Grös DF, Antony MM, Simms LJ, McCabe RE. Psychometric properties of the State-Trait Inventory for Cognitive and Somatic Anxiety (STICSA): Comparison to the State-Trait Anxiety Inventory (STAI). Psychol Assessment,. 2007;19(4):369–81.

54. Reiss S, Peterson RA, Gursky DM, McNally RJ. Anxiety sensitivity, anxiety frequency and the prediction of fearfulness. Behav Res Ther. 1986;24(1):1–8.

55. Health C for CM. Counseling Center Assessment of Psychological Symptoms (CCAPS) 2010 User Manual. 2010.

56. Tambs K, Moum T. How well can a few questionnaire items indicate anxiety and depression? Acta Psychiatr Scand. 1993;87(5):364–7.

57. Gratz KL, Roemer L. Multidimensional Assessment of Emotion Regulation and Dysregulation: Development, Factor Structure, and Initial Validation of the Difficulties in Emotion Regulation Scale. J Psychopathol Behav Assess. 2004;26(1):41–54.
